# High-Throughput All-Optical Analysis of Synaptic Transmission and Synaptic Vesicle Recycling in *Caenorhabditis elegans*

**DOI:** 10.1371/journal.pone.0135584

**Published:** 2015-08-27

**Authors:** Sebastian Wabnig, Jana Fiona Liewald, Szi-chieh Yu, Alexander Gottschalk

**Affiliations:** Buchmann Institute for Molecular Life Sciences (BMLS) and Institute of Biochemistry, Goethe-University, Max-von-Laue-Str. 9 and 15, 60438, Frankfurt, Germany; University of Edinburgh, UNITED KINGDOM

## Abstract

Synaptic vesicles (SVs) undergo a cycle of biogenesis and membrane fusion to release transmitter, followed by recycling. How exocytosis and endocytosis are coupled is intensively investigated. We describe an all-optical method for identification of neurotransmission genes that can directly distinguish SV recycling factors in *C*. *elegans*, by motoneuron photostimulation and muscular RCaMP Ca^2+^ imaging. We verified our approach on mutants affecting synaptic transmission. Mutation of genes affecting SV recycling (*unc-26* synaptojanin, *unc-41* stonin, *unc-57* endophilin, *itsn-1* intersectin, *snt-1* synaptotagmin) showed a distinct ‘signature’ of muscle Ca^2+^ dynamics, induced by cholinergic motoneuron photostimulation, i.e. faster rise, and earlier decrease of the signal, reflecting increased synaptic fatigue during ongoing photostimulation. To facilitate high throughput, we measured (3–5 times) ~1000 nematodes for each gene. We explored if this method enables RNAi screening for SV recycling genes. Previous screens for synaptic function genes, based on behavioral or pharmacological assays, allowed no distinction of the stage of the SV cycle in which a protein might act. We generated a strain enabling RNAi specifically only in cholinergic neurons, thus resulting in healthier animals and avoiding lethal phenotypes resulting from knockdown elsewhere. RNAi of control genes resulted in Ca^2+^ measurements that were consistent with results obtained in the respective genomic mutants, albeit to a weaker extent in most cases, and could further be confirmed by opto-electrophysiological measurements for mutants of some of the genes, including synaptojanin. We screened 95 genes that were previously implicated in cholinergic transmission, and several controls. We identified genes that clustered together with known SV recycling genes, exhibiting a similar signature of their Ca^2+^ dynamics. Five of these genes (*C27B7*.*7*, *erp-1*, *inx-8*, *inx-10*, *spp-10*) were further assessed in respective genomic mutants; however, while all showed electrophysiological phenotypes indicative of reduced cholinergic transmission, no obvious SV recycling phenotypes could be uncovered for these genes.

## Introduction

Chemical synaptic transmission occurs at synaptic contacts, where a presynaptic neuron releases small neurotransmitters by regulated fusion of synaptic vesicles (SVs)[1–3]. SVs consist of membrane and a specific set of proteins [4–6] that are synthesized in the neuronal soma and transported to the neuronal terminal. In the terminal, SVs undergo a constant cycle: 1) After clathrin-mediated budding from endosomes [7], they are acidified and filled with transmitter by specific transporters using the proton gradient. 2) SVs can then be directly used for fusion, or they enter a pool of vesicles that are stored for future release [8, 9]. 3) Release involves approximation of the SV to the synaptic active zone membrane, and formation of protein complexes (e.g. SNARE complexes) that regulate and execute the fusion event, processes termed docking and priming [10]. 4) SV fusion and transmitter release is triggered by neuronal depolarization and activation of voltage-gated Ca^2+^ channels, as Ca^2+^ is bound by synaptotagmin. This SV protein interacts with the SNARE complex as well as the active zone membrane to trigger the actual fusion event [11–13]. 5) Following SV fusion, proteins and membrane of the SV need to be retrieved and recycled for further rounds of transmitter release [14, 15]. Retrieval can occur by different types of endocytosis, depending on the rate of SV fusion [16]. Proteins like endophilin and synaptojanin, as well as the actin cytoskeleton and dynamin are involved in invaginating the membrane and in finally pinching it off the plasma membrane [14, 17–19]. Particularly the process of SV recycling is still enigmatic. For example, it is presently unclear how SV exo- and endocytosis are coupled [20]. Endocytosis may be triggered by the lateral pressure evoked in the plasma membrane upon SV fusion, and this may involve specific factors that sense membrane curvature or lateral pressure, and then trigger the endocytosis machinery. Exo- and endocytosis were also speculated to be coordinated based on the elevated Ca^2+^ level during neuronal activation, but such Ca^2+^ sensors have not been identified as yet [21, 22]. It is also unclear how the SV proteins are sorted such that the new SV is equipped with the right complement of proteins, at endosomes or even the plasma membrane [20]. Conceivably, specific proteins are involved in regulating these processes, but such proteins have not been described yet. Absence of these proteins should result in a shortage of functional SVs, which should become limiting particularly during sustained SV release at high rates, and this phenotype, indicative of SV recycling factors, could be used in screening approaches for such proteins.

A powerful model system for the identification and analysis of proteins associated with the SV cycle is the nematode *Caenorhabditis elegans*. Mutations in the SV release machinery lead to characteristic phenotypes of uncoordinated locomotion or paralysis [23]. In pharmacological assays using the acetylcholine esterase blocker aldicarb, which leads to progressive paralysis, reduced synaptic activity causes aldicarb resistance, as less ACh builds up in the cleft [24]. Based on this assay, RNAi screening a preselected set of ~ 2000 candidates uncovered 185 genes that may be involved in cholinergic synaptic transmission [25]. A similar screen, based on aldicarb hypersensitivity, was performed for genes potentially affecting GABAergic transmission [26]. To distinguish between pre- and postsynaptic defects, further pharmacological assays can be used. Additionally, synaptic transmission genes in *C*. *elegans* have been analyzed using fluorescence microscopy [26] and behavioral assays [27]. However, those approaches cannot distinguish between general synaptic transmission defects and defects specifically in synaptic vesicle recycling, as defects both in the biogenesis or the fusion of SVs cause the same phenotypes in these end-point assays [24, 25, 27].

An assay that can uncover failures in regeneration of SVs is based on optogenetics. Expressing and photostimulating the light-gated cation channel ChR2 in cholinergic as well as in GABAergic motoneurons is a potent tool to acutely trigger transmitter release in these neurons [28]. The evoked phenotypes, muscle contraction or relaxation, provide a temporally resolved readout for sustained transmitter release. Importantly, SV recycling mutants exhibit a progressive fatigue phenotype in ChR2 hyperstimulation assays that allows distinguishing them from general synaptic mutants [14, 28]. Thus, such behavioral assays may be used as a screening method, before more tedious electrophysiological measurements are carried out for in-depth analysis. The behavioral prescreening method was further improved using microfluidic devices and automatization to gain higher throughput [29]. However, this method is complicated by the need for microfluidic devices and experimental difficulties based on the behavioral assay. Thus an alternative readout for neuronal activity would be beneficial. Such a readout for cholinergic neuron activity could be the evoked increase in Ca^2+^ levels in the postsynaptic body wall muscle.

Combining Ca^2+^ imaging using **g**enetically **e**ncoded **c**alcium **i**ndicators (GECIs) with parallel neuronal stimulation by ChR2 is problematic due to the overlap of the excitation wavelength of both actuator and GFP-based GECIs, and, if at all possible, necessitates either selective illumination of the respective cells, or careful choice of light intensities [30–35]. Such approaches were mainly possible for experiments investigating cell to cell communication in single animals, but not for a mass readout of many animals [36, 37]. The recently established red fluorescent GECI RCaMP, however, has an excitation wavelength (maximum 590 nm) that allows imaging without concomitant ChR2 stimulation [38, 39].

In the present study, we established a new readout system for synaptic transmission in *C*. *elegans*, combining functional imaging of RCaMP in muscle of ~1000 animals and cholinergic neuron photostimulation. To allow for genetic screening using RNAi, we generated a strain with RNAi sensitization specifically only in cholinergic motoneurons in order to avoid lethality from loss of the respective proteins during development, or in other tissues. We then performed an RNAi screen of 106 genes (including positive and negative controls), 95 of which were previously identified as defective in cholinergic synaptic transmission [25], with the goal to identify SV recycling factors, that would show similar phenotypes like several known SV recycling or endocytosis factors included in the screen. Many genes showed similar phenotypes like the positive controls, based on hierarchical clustering of the data, and 5 genes were further studied. While their genomic mutants exhibited electrophysiological phenotypes, no strong SV recycling defects were obtained. In sum, our method is able to distinguish recycling factors from other proteins involved more ‘generally’ in synaptic transmission. Yet, in a possible genetic screen across the whole genome it will likely only identify genes with strong phenotypes.

## Materials and Methods

### *C*. *elegans* strains

Mutant strains used: **CB113:** *unc-17(e113)*, **CB234:** *unc-18(e234)*, **CB307:** *unc-47(e307)*, **CB406:** *unc-57(e406)*, **CB407:** *unc-49(e407)*, **CB1091:** *unc-13(e1091)*, **CB1199:** *unc-41(e1199)*, **DM612:** *unc-2(ra612)*, **EG3027:** *unc-26(s1710)*, **MT6490:** *unc-25(n2569)*, **NM204:** *snt-1(md290)*, **NM467:** *snb-1(md247)*, **NM1568:** *ehs-1(ok146)*, **RB700:** *erp-1(ok462)*, **RB2051:** *inx-10(ok2714)*, **RB2198:** *C27B7*.*7(ok2978)*, **VC116:** *inx-8 (gk42)*, **VC201:** *itsn-1(ok268)*, **VC790:** *spp-10(gk349)*, **WM27:** *rde-1(ne219)*, **ZZ20:** *unc-38(x20)*.

Generation of transgenic strains:

To generate integrated transgenes, plasmids were injected into wild type or respective mutant animals, and the initial extrachromosomal transgenes were integrated into the genome by UV irradiation in the presence of trimethylpsoralen (TMP). Animals were outcrossed at least 4 times after integration. For the integrated transgene *zxIs52*, plasmid pmyo-3::RCaMP1h (30ng/μl) was injected into wild type (N2) animals. For *zxIs58*, a mix of plasmids pmyo-3::RCaMP1h (60ng/μl) and punc-17::ChR2(C128S)::YFP (70ng/μl) was injected into *rde-1(ne219)* animals. *zxIs59* was created by injecting a mix of punc-119::sid-1 (60 ng/μl), punc-17::rde-1 (10 ng/μl) and pmyo-2::CFP (3 ng/μl).

The following transgenic *C*. *elegans* strains were generated and/or used:

**ZX460:** *zxIs6[punc-17::ChR2(H134R)::YFP; lin-15^+^],* **ZX463:** *unc-47(e307); zxIs3[punc-47::ChR2(H134R)::yfp; lin15^+^],* **ZX497:** *unc-49(e407); zxIs6[punc-17::ChR2(H134R)::YFP; lin-15^+^],* **ZX511**: *unc-26(s1710); zxIs6[punc-17::ChR2(H134R)::YFP; lin-15^+^],* **ZX531:** *unc-47(e307); zxIs6[punc-17::ChR2(H134R)::YFP; lin-15^+^],* **ZX598:** *snt-1(n2665); zxIs3[punc-47::ChR2(H134R)::yfp; lin15^+^],* **ZX1397:** *N2; zxIs52[pmyo-3::RCaMP1h],* **ZX1489**: *unc-47(e307); zxIs6[punc-17::ChR2(H134R)::YFP; lin-15^+^]; zxIs52[pmyo-3::RCaMP1h],* **ZX1490:** *unc-49(e407); zxIs6[punc-17::ChR2(H134R)::YFP; lin-15^+^]; zxIs52[pmyo-3::RCaMP1h],* **ZX1491:** *snb-1(md247); zxIs6[punc-17::ChR2(H134R)::YFP; lin-15^+^]; zxIs52[pmyo-3::RCaMP1h],* **ZX1492:** *unc-47(e307); zxIs3[punc-47::ChR2(H134R)::YFP; lin-15^+^]; zxIs52[pmyo-3::RCaMP1h],* **ZX1493:** *snt-1(md290); zxIs3[punc-47::ChR2(H134R)::YFP; lin-15^+^]; zxIs52[pmyo-3::RCaMP1h],* **ZX1494:** *zxIs3[punc-47::ChR2(H134R)::YFP; lin-15^+^]; zxIs52[pmyo-3::RCaMP1h],* **ZX1512:** *rde-1(ne219); zxIs58[pmyo-3::RCaMP1h; punc-17::ChR2(C128S)::yfp],* **ZX1513:** *rde-1(ne219); zxIs59[punc-119::sid-1; punc-17::rde-1; pmyo-2::cfp],* **ZX1514:** *rde-1(ne219); zxIs58[pmyo-3::RCaMP1h; punc-17::ChR2(C128S)::YFP]; zxIs59[punc-119::sid-1; punc-17::rde-1; pmyo-2::CFP],* **ZX1600:** *inx-10(ok2714); zxIs6[punc-17::ChR2(H134R)::YFP; lin-15^+^],* **ZX1602:** *erp-1(ok462); zxIs6[punc-17::ChR2(H134R)::YFP; lin-15+],* **ZX1603:** *C27B7.7(ok2978); zxIs6[punc-17::ChR2(H134R)::YFP; lin-15^+^],* **ZX1604:** *spp-10(gk349); zxIs6[punc-17::ChR2(H134R)::YFP; lin-15^+^],* **ZX1605:** *unc-2(ra612); zxIs6[punc-17::ChR2(H134R)::YFP; lin-15^+^],* **ZX1606:** *unc-17(e113); zxIs6[punc-17::ChR2(H134R)::YFP; lin-15^+^],* **ZX1609:** *unc-17(e113); zxIs58[pmyo-3::RCaMP1h; punc-17::ChR2(C128S)::YFP],* **ZX1610:** *unc-2(ra612); zxIs58[pmyo-3::RCaMP1h; punc-17::ChR2(C128S)::YFP],* **ZX1611:** *inx-10(ok2714); zxIs58[pmyo-3::RCaMP1h; punc-17::ChR2(C128S)::YFP],* **ZX1613:** *erp-1(ok462); zxIs58[pmyo-3::RCaMP1h; punc-17::ChR2(C128S)::YFP],* **ZX1616:** *spp-10(gk349); zxIs58[pmyo-3::RCaMP1h; punc-17::ChR2(C128S)::YFP],* **ZX1617:** *inx-8(gk42); zxIs58[pmyo-3::RCaMP1h; punc-17::ChR2(C128S)::YFP],* **ZX1631**: *unc-26(s1710); zxIs58[pmyo-3::RCaMP1h; punc-17::ChR2(C128S)::YFP],* **ZX1632:** *unc-57(e406); zxIs58[pmyo-3::RCaMP1h; punc-17::ChR2(C128S)::YFP],* **ZX1633:** *snt-1(md290); zxIs58[pmyo-3::RCaMP1h; punc-17::ChR2(C128S)::YFP],* **ZX1634:** *unc-38(x20); zxIs58[pmyo-3::RCaMP1h; punc-17::ChR2(C128S)::YFP],* **ZX1635:** *unc-13(e1091); zxIs58[pmyo-3::RCaMP1h; punc-17::ChR2(C128S)::YFP],* **ZX1636:** *zxIs58[pmyo-3::RCaMP1h; punc-17::ChR2(C128S)::YFP],* **ZX1655:** *unc-25 (n2569); zxIs3[punc-47::ChR2(H134R)::YFP]; zxIs52[pmyo-3::RCaMP1h],* **ZX1659:** *zxIs6[punc-17::ChR2(H134R)::YFP; lin-15^+^]; zxIs52[pmyo-3::RCaMP1h],* **ZX1660:** *zxIs3[punc-47::chop-2(H134R)::yfp; lin15^+^]; zxIs52 [pmyo-3::RCaMP1h],* **ZX1795** *unc-18(e234); zxIs58[pmyo-3::RCaMP1h; punc-17::ChR2(C128S)::YFP],* **ZX1796** *unc-41(e1199); zxIs58[pmyo-3::RCaMP1h; punc-17::ChR2(C128S)::YFP],* **ZX1797** *ehs-1(ok146 zxIs58[pmyo-3::RCaMP1h; punc-17::ChR2(C128S)::YFP],* **ZX1799** *itsn-1(ok268); zxIs58[pmyo-3::RCaMP1h; punc-17::ChR2(C128S)::YFP],* **ZX1800:** *rde-1(ne219); zxIs59[punc-119::sid-1,punc-17::rde-1,pmyo-2::CFP]; zxEx847[punc-119::YFP],* **ZX1971:** *inx-8(gk42); zxIs6[punc-17::ChR2(H134R)::YFP; lin-15^+^]*.

### Molecular Biology

To express SID-1 pan-neuronally, we used plasmid punc-119::sid-1 [40] (Addgene plasmid #25811). As a pan-neuronal YFP marker, we used plasmid punc-119::YFP. This plasmid was constructed by restriction digest of plasmid punc-119::sid-1 using BamHI and PvuI, and insertion of YFP coding sequence as well as part of the 3’-UTR and vector backbone (that is also found in punc-119::sid-1) retrieved by restriction digest from plasmid pCS70 (pwdfy-2::YFP; a gift from C. Schultheis), which was derived from pPD132.102 (a gift from A. Fire). To generate plasmid punc-17::rde-1, we used plasmid pmyo-3::rde-1 [41], as a source for *rde-1* cDNA. Following double digest using PspOMI and NheI, *rde-1* was retrieved and subcloned into vector pSH122, containing the *unc-17* promoter [42], cut with NheI and NotI. For expression of RCaMP1h in body wall muscle cells, we used plasmid pmyo-3::RCaMP1h [38]. For expression of ChR2(C128S)::YFP in cholinergic neurons, we used plasmid punc-17::ChR2(C128S)::YFP [43]. For feeding RNAi of GFP or YFP, we used vector L4440::GFP (Addgene plasmid # 11335).

For genotyping of mutant *C*. *elegans* strains, these primers were used:

*spp-10(gk349)*: ornai13 (5’-TCTCGCCATGTCCTGTTTCG-3’), ornai14 (5’-TGTGCTCCAAGAGCCAAAG-3’); *inx-8 (gk42)*: ornai 23 (5’-GACAGATGGCAAGGGTTC-3’), ornai52 (5’-CACCGGGTAATTCTACTACTCG-3’); *inx-10(ok2714)*: ornai31 (5’-GCTTCTCCATTAAATCTTGTGTTG-3’), ornai32 (5’-AAATCCAGCGATGCAACA AT-3’); *erp-1(ok462)*: ornai43 (5’-TGATCACTTTGGCTTGCTTG-3’), ornai44 (5’-CCAGTAATTTCGTCCGTCG T-3’); *itsn-1(ok268)*: ornai54 (5’-GAATTGTGTCACCGACATGC-3’) ornai56 (5’-GCACAACGTCAGCAACG AG-3’).

### Imaging setup for body wall muscle Ca^2+^ fluorescence signal measurements

Fluorescence measurements were carried out on an inverted fluorescence microscope (Axio Observer, Zeiss, Germany) equipped with two high-power light emitting diodes (LEDs; 470 and 590 nm wavelength, KSL 70, Rapp Optoelektronik, Germany) coupled via a beam splitter to obtain simultaneous dual-wavelength illumination. Excitation light was passed through a double band pass excitation filter (479 and 585 nm), combined with a 605 nm beam splitter and a 647 nm emission filter (F74-423, F38-605, and F37-647, respectively; AHF Analysentechnik, Germany). Blue light illumination protocols (temporal sequences) were programmed on, and generated by, a Lambda SC Smart shutter controller unit (Sutter Instruments, USA), using its TTL output to drive the LED power supply. ChR2 stimulation was performed using 1.4 mW/mm^2^ blue light, unless otherwise stated. Image acquisition was performed using μManager [44]. For Ca^2+^ imaging of single animals, they were immobilized on 10% M9 agar pads with polystyrene beads (Polysciences, USA) [45].

### Preparing agar-based micro-wells for bulk Ca^2+^ fluorescence signal measurements

M9 buffer was used to dissolve 10% (w/v) agar by boiling in a microwave oven. Microscope slides were prepared according to [38]. The agar pads were generated to be thicker, by using a glass slide as spacer. A small metal LED cooler (“Stiftkühlkörper” ICK S 14X14X10 (L x W x H) 14 x 14 x 10 mm R(th) 9.8 K/W, S. Fischer Elektronik, Germany) was fixed on an aluminum plate and heated in a gas burner. The hot cooler was then punched into the agar pads to obtain reproducible micro-wells. These were checked before use for quality and consistency using a stereo microscope (**S1 Fig**).

### Preparation of animals for bulk Ca^2+^ fluorescence signal measurements

Animals were washed off the culture dish with 1 ml of M9 buffer, and collected in a 1.5 ml eppendorf tube. Animals were allowed to settle for 5–10 minutes. 60 μl of the worm “sediment” at the bottom of the tube were transferred to a tube containing 1 ml of fresh M9 buffer, then animals were centrifuged at 2800 rpm for 2 min. Micro-wells were filled with 2x 3.5μl worm pellet.

### RNAi procedure

RNAi clones were picked from the Ahringer bacterial RNAi feeding library [46] (or in few cases, the Vidal library) and grown over night on lysogeny broth (LB)—Ampicillin containing agar dishes. Colonies were picked and cultivated over night at 37°C and 180 rpm in 5 ml LB Ampicillin supplemented media. Nematode growth medium (NGM) Ampicillin plates were seeded with 200 μl culture / plate and incubated at 37°C over night. Next day, RNAi feeding bacteria were induced to synthesize dsRNA by application of 150 μl Isopropyl-β-D-thiogalactopyranosid (IPTG) stock solution (100mM), and then 15 L4 animals of the RNAi-sensitive strain ZX1514 were transferred onto those plates. A second batch of NGM-Ampicillin plates was seeded with the respective RNAi feeding strain. These were induced on the following day by 150μl (100mM) IPTG stock containing all-*trans* retinal (ATR; 4μl stock solution of 100mM/ml), and 10 adult animals from the respective first culture dish were transferred onto the new culture dish and their progeny cultivated for 4 (or 5, if growth was unusually slow) days.

### Image and data analysis

Image analysis was performed in ImageJ (NIH). Analysis of immobilized single animals was done using a region of interest (ROI) placed around the animal and a second ROI for background fluorescence. Mean intensity values for each video frame were obtained and background fluorescence was subtracted from the fluorescence values derived from RCaMP expressed in body wall muscle cells (BWMs). Subtracted data was normalized to ΔF/F_0_ = (F_i_-F_0_)/F_0_, where F_i_ represents the intensity at this time point and F_0_ represents the average of the first 20 time points during blue light stimulation. For bulk measurements videos of the whole field of view were acquired. Analysis was performed by taking the whole micro-well, filled with animals, as one ROI. Mean fluorescence values were corrected for the intensity increase during blue light stimulation. To this end, the difference between the average of the last 30 frames before, and the first 20 frames during blue light stimulation was subtracted from data points during blue light stimulation. Traces were normalized by ΔF/F_0_ = (F_i_-F_0_)/F_0_, where F_i_ represents the intensity at that time point and F_0_ the baseline as average from the first 30 frames before blue light stimulation. To take into account that peak fluorescence was not reached precisely at the same time for biological replicates, the traces were peak-normalized, then averaged, and again peak normalized, such that difference traces could be calculated between RNAi animals and the respective animals treated with empty RNAi bacteria (see **S2 Fig and S1–S3 Data**). Data analysis was carried out in either Excel (Microsoft, USA) or Prism software (GraphPad, USA). To distinguish significantly different behavior in RNAi animals compared to controls, we used 2-way ANOVA, with Bonferroni correction, across the entire time course, for the 3–5 replicates of the experiments for each gene, and the respective controls analyzed on the same day. An R script to generate color-coded graphs was kindly provided by C. Schultheis. Hierarchical clustering was performed using the Excel add-in collection XLSTAT (Addinsoft, France). Clustering was done using agglomerative hierarchical clustering (AHC), with dissimilarity determined as Manhattan distances, with complete linkage, and defining 6 classes.

### Quantification of nerve cord YFP expression

To quantify YFP expression we imaged animals fed with “empty” RNAi feeding vector, L4440, versus those fed with the respective anti-GFP RNAi dsRNA producing bacteria (likewise targeting YFP), using identical microscope settings. We took the background-corrected absolute fluorescence values over the whole ventral nerve cord, and averaged this for several animals. To image and assign individual ventral cord neurons (i.e. GABAergic and cholinergic motoneurons), we imaged the animals under a spinning disk confocal microscope (Zeiss Cell Observer SD) and produced maximum projection z-stacks, again using identical microscope settings for control strain and GFP-RNAi animals.

### Electrophysiological measurements

Electrophysiological recordings from dissected BWMs were essentially performed as described previously [47]. After dissection, cells were treated for 8s with 0.5 mg/ml collagenase (Sigma, Germany) in *C*. *elegans* Ringer’s (CRG; 150mM NaCl, 5mM KCl, 5mM CaCl_2_, 1mM MgCl_2_, 10mM glucose, 15mM HEPES (pH 7.35), 340mOsm) and washed with CRG. Cells were clamped to a holding potential of -60mV using an EPC10 amplifier with head stage and Patchmaster software (HEKA, Germany). The bath solution was CRG, the pipette solution was 115 mM K-gluconate, 25 mM KCl, 0.1 mM CaCl_2_, 5 mM MgCl_2_, 1 mM BAPTA, 10 mM HEPES, 5 mM Na_2_ATP, 0.5 mM Na_2_GTP, 0.5 mM cAMP, 0.5 mM cGMP, pH 7.2, with ∼320 mOsm KOH. Light activation (10 ms pulses, 0.5 Hz) was performed using an LED lamp at a wavelength of 470nm (KSL-70, Rapp OptoElectronic, Germany; 8 mW/mm^2^) and controlled by Patchmaster software (HEKA, Germany). For data analysis MiniAnalysis (Synaptosoft, USA) and Origin (OriginLab, USA) software were used.

## Results

### A Ca^2+^ imaging assay capable of detecting synaptic transmission defects

**Fig 1:**
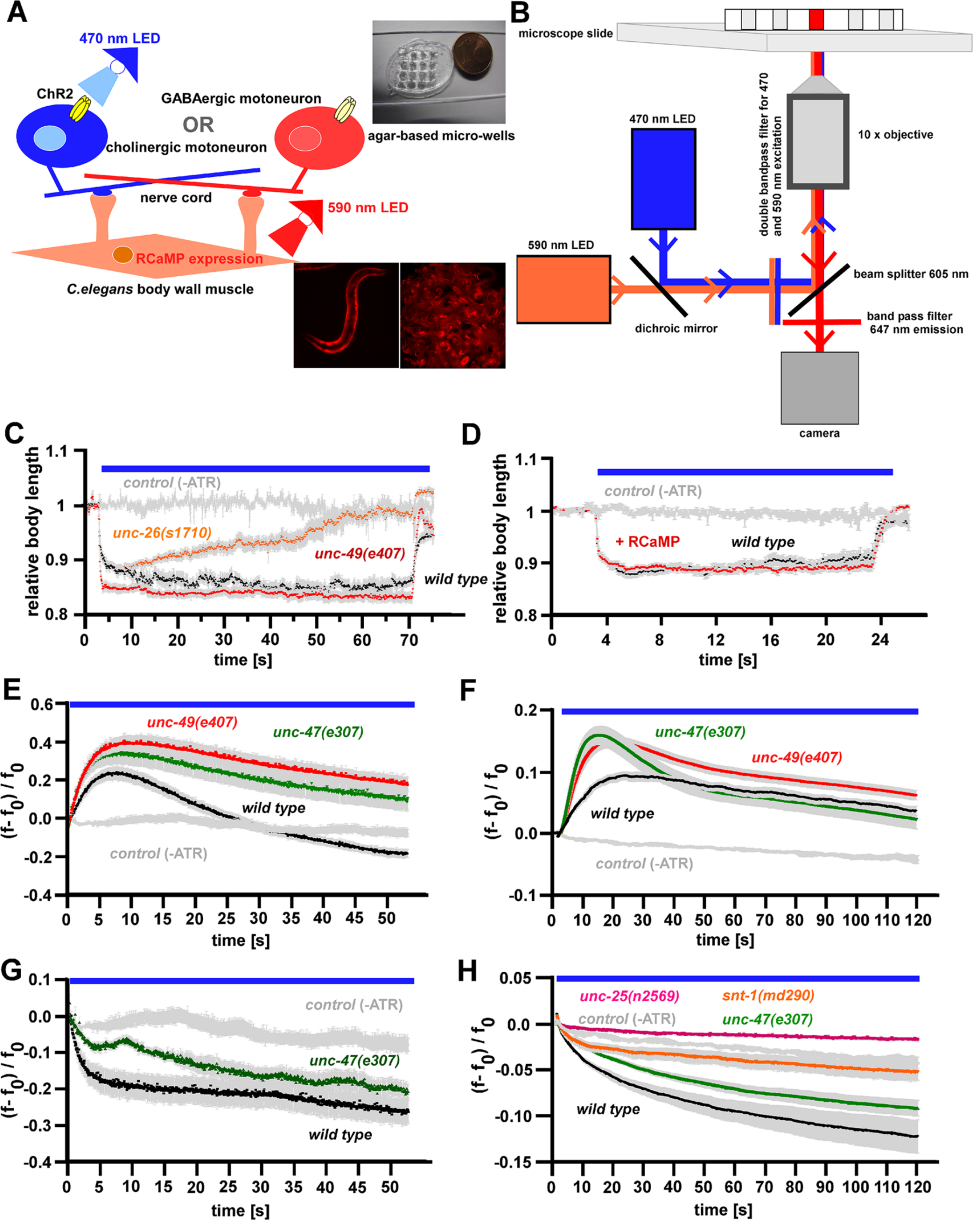
Establishing the R-OptIoN approach. **A)** Schematic of the Ca^2+^ imaging approach showing single animal and bulk measurement conditions (lower inset, left and right, respectively, showing RCaMP fluorescence in BWMs). The 470 and 590 nm LEDs were used to stimulate ChR2 expressed in neurons, and for exciting RCaMP expressed in BWMs, respectively. The micro-wells (upper inset) were prepared in a thick 10% agar pad, pierced with a heated LED cooler element. **B)** Schematic of the epifluorescence microscope used for Ca^2+^ imaging. Two high power LEDs are coupled into the excitation light train with a dichroic mirror; light passes through a 470/593 nm double band-pass excitation filter and a 605 nm beam splitter onto the animals in the micro-wells. Emission light passes a 647 nm filter before reaching the camera. **C)** Comparison of changes in body length of animals expressing ChR2 in cholinergic motoneurons in wild type background (black trace), *unc-49(e407)* (GABA_A_R) mutants (red trace), or *unc-26(s1710)* (synaptojanin) mutants (orange trace). In the absence of ATR, rendering ChR2 non-functional, no contraction is observed (grey trace). Shown is the mean normalized body length (± SEM; n = 8–21). Blue bar marks period of illumination. **D)** Light-induced activation of cholinergic neurons expressing ChR2 causes essentially identical changes in body length (contraction) with and without RCaMP expression in BWMs (red and black trace, respectively). In the absence of ATR, no contraction is observed (grey trace). Shown is the mean normalized body length (± SEM; n = 11–27 animals). **E)** Ca^2+^ response in BWMs of single animals fixed on polystyrene beads during photostimulation of ChR2 expressed in cholinergic motoneurons. Displayed are mean ΔF/F_0_ values (± SEM) of wild type control (black trace), *unc-47(e307)* mutants (green trace), and *unc-49(e407)* mutants (red trace) of n = 5–15 animals. **F)** Ca^2+^ response in BWMs during photostimulation of ChR2 expressed in cholinergic motoneurons in bulk measurements. Displayed are mean ΔF/F_0_ values (± SEM) of wild type control (black trace), *unc-47(e307)* mutants (green trace), and *unc-49(e407)* mutants (red trace). Shown is the mean of n = 3 measurements of ~ 1000 animals each. **G)** Ca^2+^ response in BWMs of single animals fixed on polystyrene beads during photostimulation of ChR2 expressed in GABAergic motoneurons. Displayed are mean ΔF/F_0_ values (± SEM) of wild type control (black trace), *unc-47(e307)* mutants (green trace) and no-ATR control (grey trace), of n = 6–9 animals for each genotype/condition. **H)** Ca^2+^ response in BWMs during photostimulation of ChR2 expressed in GABAergic motoneurons in bulk measurements. Displayed are mean ΔF/F_0_ values (± SEM) of wild type control (black trace), *snt-1(md290)* mutants (orange trace), *unc-47(e307)* mutants (green trace), and *unc-25(n2569)* mutants (magenta trace); means ± SEM of n = 3 measurements of ~1000 animals each.

To monitor synaptic transmission in an all-optical manner, we expressed RCaMP in body wall muscle cells (BWMs) combined with ChR2 in cholinergic or GABAergic motoneurons (**Fig 1A**), enabling simultaneous ChR2 induced neuronal hyperstimulation and Ca^2+^ imaging [38]. RCaMP excitation (590 nm) and readout (647 nm) combined well with ChR2 stimulation (470 nm) using two LEDs, on a standard inverted fluorescence microscope equipped with a sCMOS camera (**Fig 1B**). As shown previously, photostimulation of cholinergic motoneurons in different mutant backgrounds results in specific contraction profiles over time [14, 28]. In this assay, mutations generally affecting SV release exhibit stronger contraction than wildtype, which is due to compensatory higher excitability in muscle [28], and SV recycling mutants (like in *unc-26* synaptojanin or *unc-57* endophilin) show a progressively decreasing contraction, as SVs become depleted (**Fig 1C**). Mutants affecting GABA signaling (like the post-synaptic GABA_A_ receptor *unc-49*) similarly show stronger contractions. We verified that expression of RCaMP did not alter BWM physiology, as contractions caused by ChR2 stimulation of cholinergic motoneurons were normal (**Fig 1D**). We next performed Ca^2+^ imaging experiments in animals in which cholinergic neurons were stimulated for 60s in wild type or mutant backgrounds lacking GABAergic inhibition (*unc-49(e407)* GABA_A_R and *unc-47(e307)* pre-synaptic vesicular GABA transporter (vGAT); **Fig 1E**). Muscle Ca^2+^ signals reached a maximum (~20% ΔF/F_0_) within 7 s, then declined within the next 20 s (**Fig 1E**); in control animals, cultivated without the ChR2 chromophore all-*trans* retinal (ATR), no obvious change in the Ca^2+^ signal occurred. To obtain more robust data, particularly during an RNAi screen, we used small wells in an agarose matrix (**Fig 1A; S1 Fig**), enabling simultaneous imaging of ~1000 tightly packed animals, which can be imaged essentially in the same focal plane, using a low magnification (10x) objective (**Fig 1A and 1B; S3 Fig**). When cholinergic neurons were photostimulated in such mass analyses for 120 s, muscle Ca^2+^ signals reached a ~10% maximum ΔF/F_0_ within 20 s, and declined during the next 100 s (**Fig 1F; S3 Fig**); in animals cultivated without ATR, no obvious Ca^2+^ signals were seen. When stimulating GABAergic motoneurons, Ca^2+^ signals dropped, both in single animal or mass measurements (~20 or ~10% ΔF/F_0_, respectively; **Fig 1G and 1H**). Consequently, in *unc-47(e307)* vGAT mutants, or in *unc-49(e407)* GABA_A_R mutants, higher Ca^2+^ increases, or smaller Ca^2+^ decreases were obtained upon cholinergic or GABAergic motoneuron stimulation, respectively (**Fig 1E–1H**). This is consistent with more pronounced contraction upon cholinergic neuron stimulation (**Fig 1C**). In mutants lacking all GABA (*unc-25(n2569)*, encoding glutamic acid decarboxylase—GAD), no obvious reduction in muscle Ca^2+^ signal was observed upon 120 s GABA neuron stimulation (**Fig 1H**). In *snt-1(md290)* mutants of synaptotagmin, required for SV exocytosis and recycling [28, 48] a less-pronounced Ca^2+^ decrease was seen upon GABAergic stimulation (**Fig 1H**), in agreement with our previous electrophysiological analysis [28]. We thus term this method R-OptIoN (RCaMP-mediated Optogenetic Investigation of Neurotransmission).

### Establishing bulk Ca^2+^ measurements for high-throughput screening

**Fig 2:**
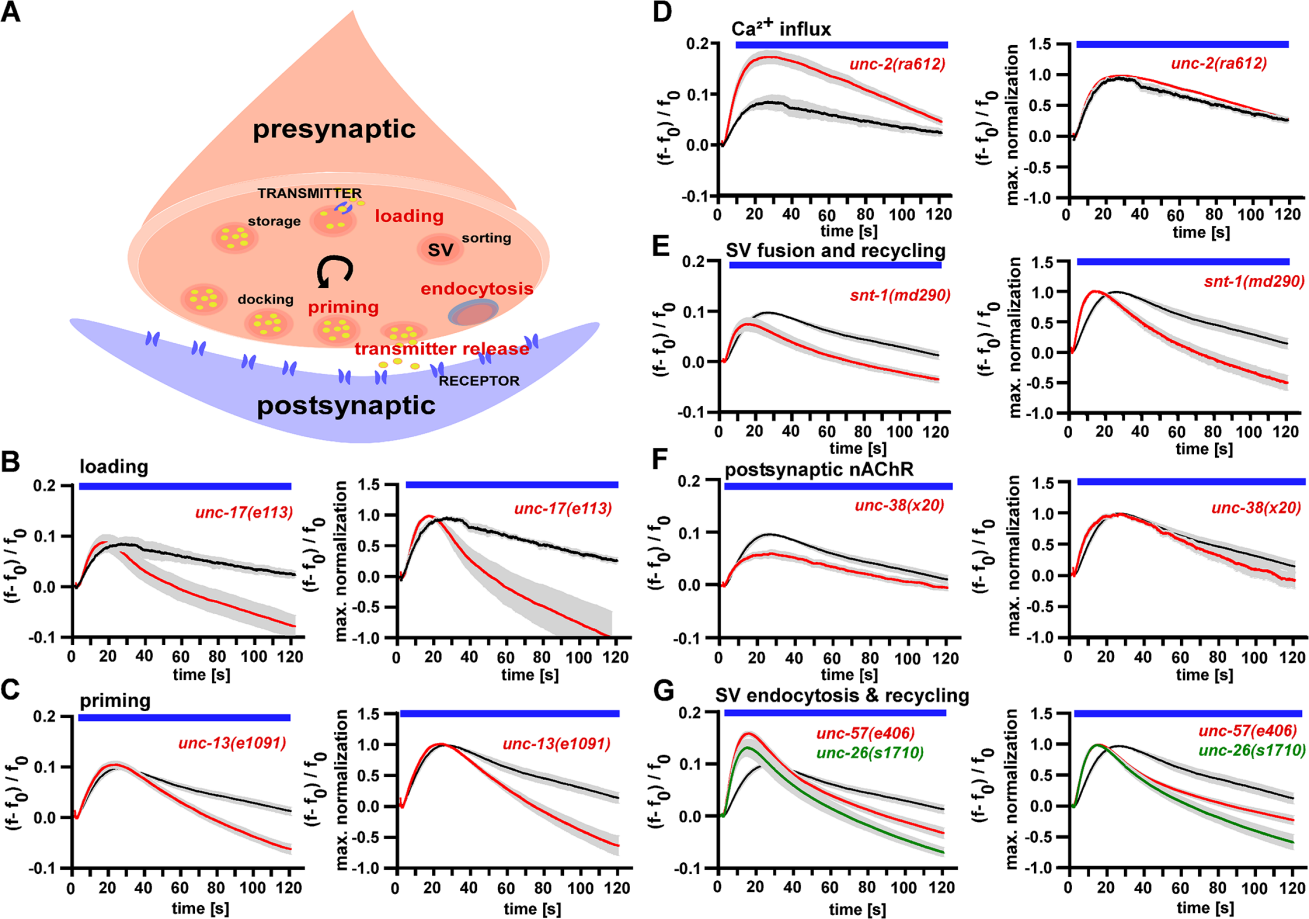
R-OptIoN analysis of mutants that had previously been described to affect the synaptic vesicle cycle [25, 26]. **A)** Schematic representation of the synaptic vesicle cycle, showing (anti-clockwise) loading of SV with transmitter, transport of SV to the plasma membrane, docking, priming into the fusion-competent state, SV-plasma membrane fusion upon depolarization and Ca^2+^ influx, endocytosis/recycling of SV membrane and proteins, biogenesis of SV from endosomal compartment. **B-G)** Ca^2+^ traces resulting from bulk measurements in animals expressing ChR2(C128S) in cholinergic motoneurons and RCaMP in BWMs (displayed are means ± SEM of n = 3 experiments, ~1000 animals each). The blue bar marks the period of illumination. Graphs on the left describe ΔF/F_0_ Ca^2+^ dependent RCaMP fluorescence signals, graphs on the right side show maximum normalized ΔF/F_0_ Ca^2+^ response. The analyzed mutants affect different steps in the SV cycle such as SV loading (**B**; *unc-17(e113)*), priming (**C;** *unc-13(e1091)*, Ca^2+^ influx (**D;** *unc-2(ra612)*, VGCC), SV fusion and recycling (**E;** *snt-1(md290)*), postsynaptic defects in an AChR subunit (**F;** *unc-38(x20)*) and SV recycling (**G;** *unc-26(s1710)* synaptojanin and *unc-57(e406)* endophilin A).

**Fig 3:**
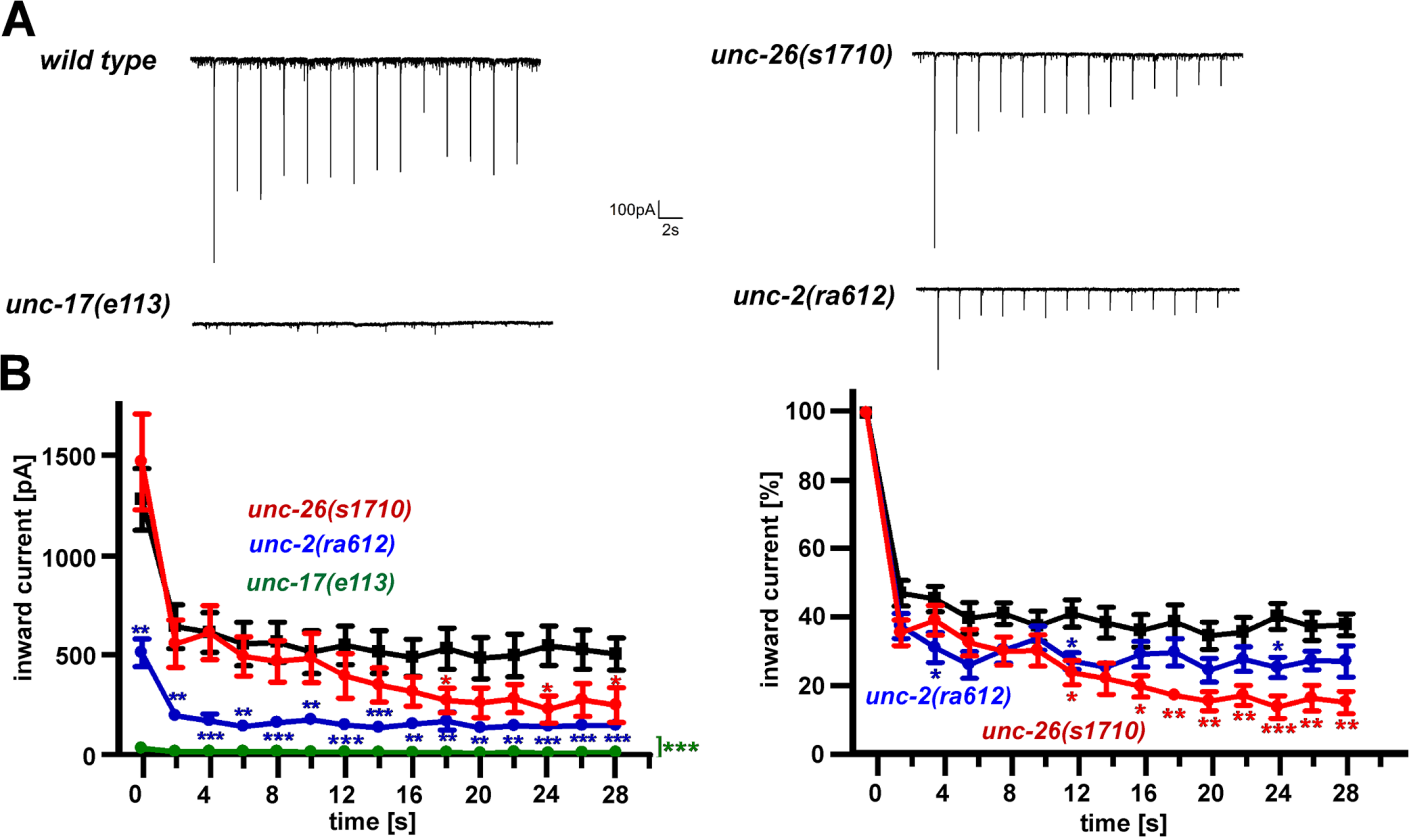
Electrophysiology verifies SV loading, release and recycling phenotypes. Photoevoked excitatory post-synaptic currents were measured in patch-clamped muscle, during repeated photostimulation of cholinergic neurons expressing ChR2, at 0.5 Hz. **A)** Representative original current traces of wild type, compared to mutants affecting SV loading (*unc-17(e113)* vAChT), SV recycling (*unc-26(s1710)* synaptojanin, or Ca^2+^ influx (*unc-2(ra612)* VGCC). **B)** Group data and statistical analysis, corresponding to the experiments in A. Wild type data is shown in black. Left panel: Mean inward currents (pA) ± SEM, in response to repeated stimulation with 10 ms blue light at 0.5 Hz. Right panel: Data was normalized to the first peak inward current [%]. Statistically significant differences were assessed by one-way ANOVA compared to wild type (* P < 0.05, ** P < 0.01, *** P < 0.001). For analysis of miniature PSC currents and frequencies of these strains, see **S5 Fig.**

To evaluate whether our all-optical system for analyzing synaptic transmission defects will enable assessing the likely nature of the defect (particularly ‘general’ synaptic release vs. SV recycling) in bulk Ca^2+^ measurements in BWMs during stimulation of cholinergic motoneurons, we used different mutants affecting the synaptic vesicle cycle (**Fig 2A**). We wanted to compare observed Ca^2+^ responses and our previous behavioral and electrophysiological characterizations [28]. As we image a large number of animals ‘pelleted’ into micro-wells, light scattering or absorption may reduce photostimulation effects. We thus chose the ChR2 variant C128S which provides higher light sensitivity compared to ChR2(H134R) [43]. The permanent simultaneous 590 nm illumination during stimulation resulted in equivalent contraction values for ChR2(C128S) and ChR2(H134R) (**S4 Fig**; compare to **Fig 1C**). Next we compared Ca^2+^ traces of several mutants in their response to cholinergic motoneuron stimulation with the wild type (**Fig 2B–2G**). Fluorescence was analyzed as ΔF/F_0_ after correcting for the baseline shift induced by the ChR2 stimulation blue-light (**Fig 2B–2G**, left panels; **S2 Fig**), averaging fluorescence from the whole micro-well (**S1 Video**). This allowed comparing the extent of signaling from neuron to muscle, and possibly also reflected muscle excitability. In addition, to allow a better comparison of Ca^2+^ dynamics, we normalized the data of mutant and wild type traces to the peak values (**S2 Fig; Fig 2B–2G**, right panels). The corresponding peak-normalized Ca^2+^ traces of mutant and wild type were compared by 2-way ANOVA with Bonferroni correction, to identify significantly different Ca^2+^ dynamics; all genes analyzed in **Fig 2B–2G** met this criterion. Impairments in SV loading (*unc-17(e113)*; vesicular acetylcholine transporter, vAChT [49]), or in SV priming (*unc-13(e1091)* [50–52]) did not affect peak Ca^2+^ increases, while peak-normalized traces showed an earlier fatigue (**Fig 2B and 2C**). Impairment of pre-synaptic Ca^2+^ signaling (*unc-2(ra612)*; voltage-gated Ca^2+^ channel, VGCC [53]), caused an overall increased Ca^2+^ response, with otherwise normal Ca^2+^ dynamics (**Fig 2D**). Defective transmitter release (*snt-1(md290)*; synaptotagmin [54, 55]), as well as defective post synaptic transmitter sensing (*unc-38(x20)*; nicotinic acetylcholine receptor subunit, nAChR [56, 57]) led to an overall weaker Ca^2+^ response (**Fig 2E and 2F**). Endocytosis defects, impairing SV recycling (*unc-57(e406)* endophilin, *unc-26(s1710)* synaptojanin [14, 17, 58]), led to a stronger and faster (0–20 s) onset in muscle Ca^2+^ signals during long-term stimulation compared to wild type animals (**Fig 2G**). This was accompanied by a faster and earlier (> 20 s) appearing fatigue, as compared to other pre- or post-synaptic mutations, apparent also in contraction assays (**Fig 1C; S4 Fig**). We also analyzed these mutants in electrophysiological assays. Cholinergic neurons were photostimulated at 0.5 Hz, and post-synaptic currents were recorded (**Fig 3A and 3B**). This showed largely reduced or essentially abolished post-synaptic currents in *unc-2(ra612)* or *unc-17(e113)* mutants, respectively. For *unc-26(s1710)* synaptojanin mutants, currents were progressively getting smaller, demonstrating the SV recycling deficit in these animals.

Why some pre-synaptically compromised mutants even show a more pronounced and/or faster rise in post-synaptic Ca^2+^ is not clear. Previously we showed that in *snb-1* and *unc-13* mutants, which release very little ACh (based on postsynaptic patch clamp measurements of evoked release), muscle cells exhibit a strongly increased excitability, probably as a compensatory effect [28]. Importantly, the ‘defective SV endocytosis / recycling signature’, i.e. stronger and faster onset and earlier fatigue of Ca^2+^ signals, represents a distinctive feature that may well serve to identify such mutants. For example, consistent with its additional role in endocytosis, faster onset and earlier fatigue were also observed in the synaptotagmin mutant (**Fig 2E**).

### Selective RNAi of genes in cholinergic neurons facilitates reverse genetic screening

**Fig 4:**
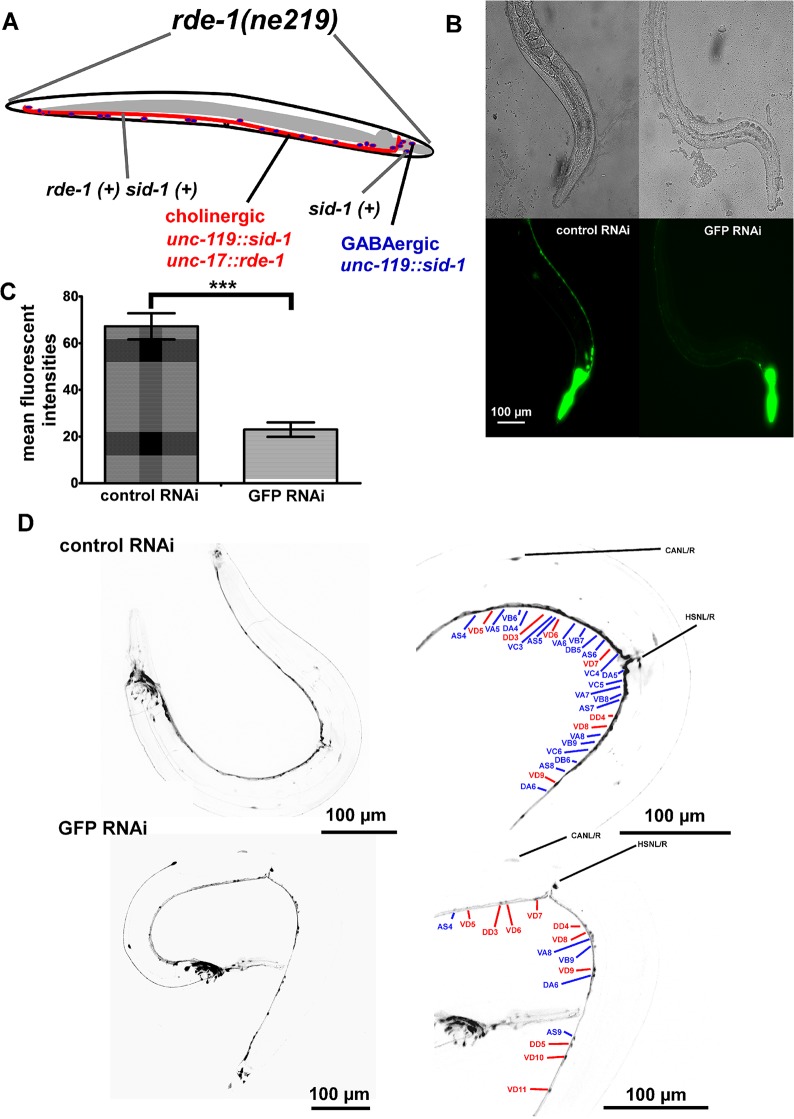
Construction and characterization of a strain with specific RNAi sensitivity in cholinergic motoneurons. **A)** The genetic background lacks *rde-1*, eliminating RNAi in all tissues. RDE-1 is selectively rescued in cholinergic neurons, by expression from the *unc-17* promoter. Furthermore, neurons are sensitized to RNAi by overexpression of the SID-1 dsRNA uptake facilitator (using the panneuronal *unc-119* promoter). **B)** Specific knockdown (feeding RNAi) of YFP in cholinergic neurons (using GFP-RNAi bacteria that also target YFP), in an animal expressing YFP panneuronally (punc-119::YFP). Left, mock-RNAi control; right, YFP knockdown, residual YFP fluorescence in ventral nerve cord mainly from GABAergic neurons. **C)** Group data and statistics of animals as shown in B. Mean ± SEM, background-corrected fluorescence values resulting from the whole nerve cord YFP fluorescence. Compared are animals treated with mock-RNAi vector (L4440) (n = 21) or with L4440::GFP RNAi (n = 15), showing statistically significant reduction of YFP fluorescence (t-test, *** P<0.001). **D)** Pan-neuronal YFP expression in the RNAi-sensitive strain ZX1800. Animals were treated with bacteria containing the mock-control (L4440) (upper images), or (lower images) the GFP RNAi vector. Reduced YFP expression in the ventral nerve cord can be recognized. In the right images, cholinergic (blue) and GABAergic (red) motoneurons are labeled, in maximum projections of confocal stacks; shown is an enlarged region of the ventral nerve cord surrounding the vulva. Anti-GFP RNAi renders most cholinergic neurons undetectable due to YFP mRNA knock-down.

RNAi can be conveniently achieved in *C*. *elegans* by feeding *E*. *coli* expressing double-stranded RNA homologous to the mRNA of interest [59, 60]. However, to make neurons susceptible to RNAi, certain mutant backgrounds have to be used [40, 61, 62]. Systemic RNAi by feeding can cause lethality or larval arrest if the gene is needed during development, even though the gene may not be essential in adult animals (while still being required for function of the cells of interest). Cholinergic cells are essential for survival, but mutations affecting synaptic recycling do not necessarily cause lethal loss of function in these neurons, while they may have lethal effects if absent in other tissues. Thus, to facilitate reverse genetic screening in cholinergic motoneurons based on the ChR2-RCaMP combination, we generated a strain for enhanced neuronal RNAi in just these neurons. This has been similarly demonstrated previously for GABAergic neurons [63]. To this end, we over-expressed SID-1 pan-neuronally (which facilitates uptake of dsRNA) [40]. We used *rde-1(ne219)* mutants that are defective in RNAi [64], and rescued RDE-1 specifically in cholinergic neurons (**Fig 4A**). To verify that our RNAi strain allowed cell-type specific RNAi, we expressed YFP pan-neuronally, and fed RNAi bacteria specific for GFP (which also target YFP). Compared to transgenic control animals, YFP expression in the nerve cord was significantly reduced by 66% (**Fig 4B and 4C**). This reduction could be attributed to cholinergic motoneurons, and their processes, which are representing ~75% of the cell bodies in the ventral nerve cord (56 cholinergic vs. 19 GABAergic motoneurons). To assess whether RNAi treatment decreased YFP expression specifically in cholinergic motoneurons, we used confocal imaging (**Fig 4D**). Almost no cholinergic cell bodies, but all GABAergic cell bodies in the nerve cord could be detected, while in control animals, YFP expression was strong in all nerve cord neurons, including the numerous cholinergic cell bodies and neuron processes.

### Analyzing synaptic mutants by RNAi in cholinergic neurons and postsynaptic RCaMP imaging

**Fig 5:**
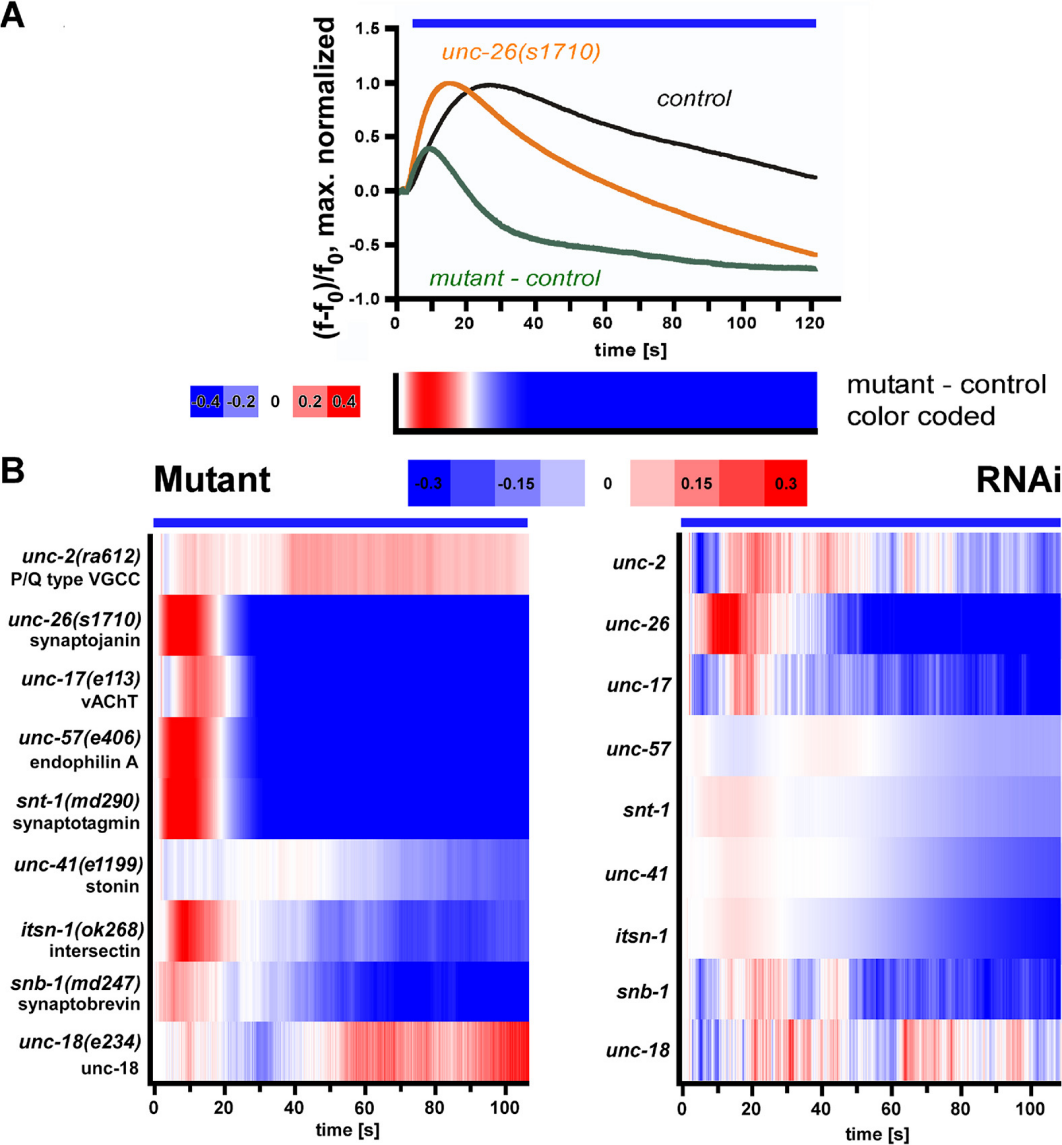
Representation of Ca^2+^ imaging data for high throughput analyses and recapitulation of mutant phenotypes by cholinergic neuron RNAi. **A)** Maximum-normalized ΔF/F_0_ Ca^2+^ signals from bulk measurements, before and during 120 s cholinergic neuron ChR2 photostimulation, in wild type control (black trace), and *unc-26(s1710)* synaptojanin mutant (orange trace). The difference graph (wild type—mutant trace) is shown in green. Mean of n = 3 experiments with ~1000 animals each. The difference trace is further plotted in color-code below the graph, color scale is shown on the left. Blue bar marks the period of illumination. **B)** Comparing color-coded Ca^2+^ signal difference traces from bulk measurements in genomic mutants (left panel) and respective RNAi animals (right panel); genotypes or mRNAs targeted, as indicated. Data averaged from n = 3–18 experiments with ~1000 animals each.

We next compared several mutants affecting synaptic transmission, and particularly, in endocytosis and SV recycling, in our all-optical assay, to the equivalent RNAi knockdown animals using the cholinergic neuron RNAi-sensitized strain. In order to be able to quickly assess the data, particularly for high-throughput screening, we devised a color-coded representation. To this end we subtracted the peak-normalized traces of mutant / knockdown animals from the respective wild type / control animals. The resulting difference trace is positive (shades of red) when mutant Ca^2+^ traces increase faster than wild type (as for example in *unc-26* synaptojanin SV-recycling mutants), and becomes negative (shades of blue) when mutant Ca^2+^ levels drop below wild type controls (**Fig 5A and 5B**). We compared RNAi knockdown to respective mutants for the following genes (involved in the following processes; all analyses in **Fig 5B** showed significantly different changes based on 2-way ANOVA with Bonferroni correction; see **S1 Data**): *unc-2* (VGCC, required to trigger SV fusion), *unc-26* synaptojanin (dephosphorylating phospholipid headgroups at endocytosed membranes; uncoating of clathrin coated membranes; SV recycling), *unc-57* endophilin (shaping endocytosed membranes; SV recycling), *unc-41* stonin (endocytosis sorting adaptor; recycling of synaptotagmin and SVs [65]), and *itsn-1* intersectin (coordinates endocytosis and actin assembly; SV recycling [66]). In addition, we analyzed *unc-17* vAChT (SV filling), *snt-1* synaptotagmin (Ca^2+^ sensor for exocytosis, and adaptor for AP2 assembly at SV fusion sites; SV recycling), *snb-1* synaptobrevin (SNARE protein required for fusion), and *unc-18* (SV priming factor, limiting availability of fusion-competent SVs). While *unc-2(ra612)* (and, to some extent, *snb-1(md247)*) showed inconclusive Ca^2+^ difference traces, and the period of positive Ca^2+^ signals was longer than 20 s, endocytosis/SV recycling factors *unc-26(s1710)*, *unc-57(e406)*, *snt-1(md290)* and *itsn-1(ok268)* exhibited a fast rise (0–20 s) and fast decay (> 20 s) of Ca^2+^ signals, reflected by the color-coded difference graph (**Fig 5B**, left panel). This was comparable in RNAi animals for *unc-26*, *snt-1* and *itsn-1* (**Fig 5B**, right panel), although the extent of the effects was most robust for *unc-26* RNAi. RNAi, but not mutation of the stonin *unc-41(e1199)* showed a very weak SV recycling signature, compared to *unc-26*, *unc-57*, *itsn-1* and *snt-1*, while the priming factor *unc-18(e234)* exhibited no such signature. Overall, mutant phenotypes and RNAi phenotypes matched relatively well, while the extent of the effects was mostly different (and generally stronger for the mutants). We also analyzed the *ehs-1(ok146)* mutant, encoding an Eps15 homolog (involved in SV recycling together with ITSN-1), and *dyn-1* dynamin RNAi animals, that also exhibited this signature (apart from the late time points for *ehs-1*; **S6 Fig**).

Interestingly, *unc-17(e113)* mutants, affecting the vesicular ACh transporter, showed Ca^2+^ dynamics that were similar to the SV recycling signature, i.e. a positive Ca^2+^ difference trace at early time points (5–25 s), and a decay below wild type at > 20–25 s (*unc-17* mutant and RNAi differed slightly). As the SV cycle is a circular process, it is not obvious at which stage a bottleneck, leading to progressive SV depletion, is situated. vAChT is responsible for SV filling with ACh, thus reduction of its function would likewise lead to more and more empty vesicles upon excessive synaptic activity, with the same (post-synaptic) consequence as a SV recycling defect. In contrast, a reduced or blocked activity of UNC-18(and, somewhat, SNB-1), which prime or fuse SVs, would not alter the amount of (functional) SVs available, as less SVs are fused, but also less SVs need to be recycled. So no progressively exacerbating phenotype is expected for such mutants, as seen in our assays (**Fig 5B**). In a reverse genetic screen, we expect to find both mutants affecting SV endocytosis and recycling, as well as filling and priming, and our assays may suffice for an initial distinction, if the recycling phenotype is strong. The progressive decay of transmitter release must then be further assessed by electrophysiological recording (**Fig 3A and 3B; S5 Fig**).

### An all-optical RNAi screen for genes affecting SV recycling

We used our approach to perform a pilot RNAi screen in our cholinergic neuron sensitized strain. We chose 106 genes for this screen (**S1 Table**): 11 were random picked genes with no known role in synaptic transmission. Among the other 95 genes, we included five known SV recycling factors (*unc-26*, *unc-57*, *itsn-1*, *unc-41* and *snt-1*) as well as four genes known or expected to be involved in endocytosis (*dyn-1*, *aps-2*, *apa-2*, *erp-1*) while for the remaining 86 genes, though they were previously implicated in cholinergic synaptic transmission, based on pharmacological (aldicarb resistance) assays and systemic RNAi [25], no specific role in the SV cycle was known. These included 36 genes that Sieburth et al. (2005) had verified for aldicarb resistance in genomic mutants, 45 genes which had not been verified in mutants, and one for which systemic RNAi caused lethality (*rab-5*). We reasoned that our approach of restricting RNAi to cholinergic neurons may avoid lethality and/or uncover functions specifically in cholinergic synaptic recycling. We included two genes that were shown to generate aldicarb hypersensitivity when depleted, acting in GABAergic transmission (*goa-1* Gα_o_ and *unc-43* CaMKII). Last, one postsynaptic aldicarb resistance gene (*unc-29*, a muscular nAChR subunit), and one general trafficking gene, the kinesin *unc-104*, were assessed.

**Fig 6:**
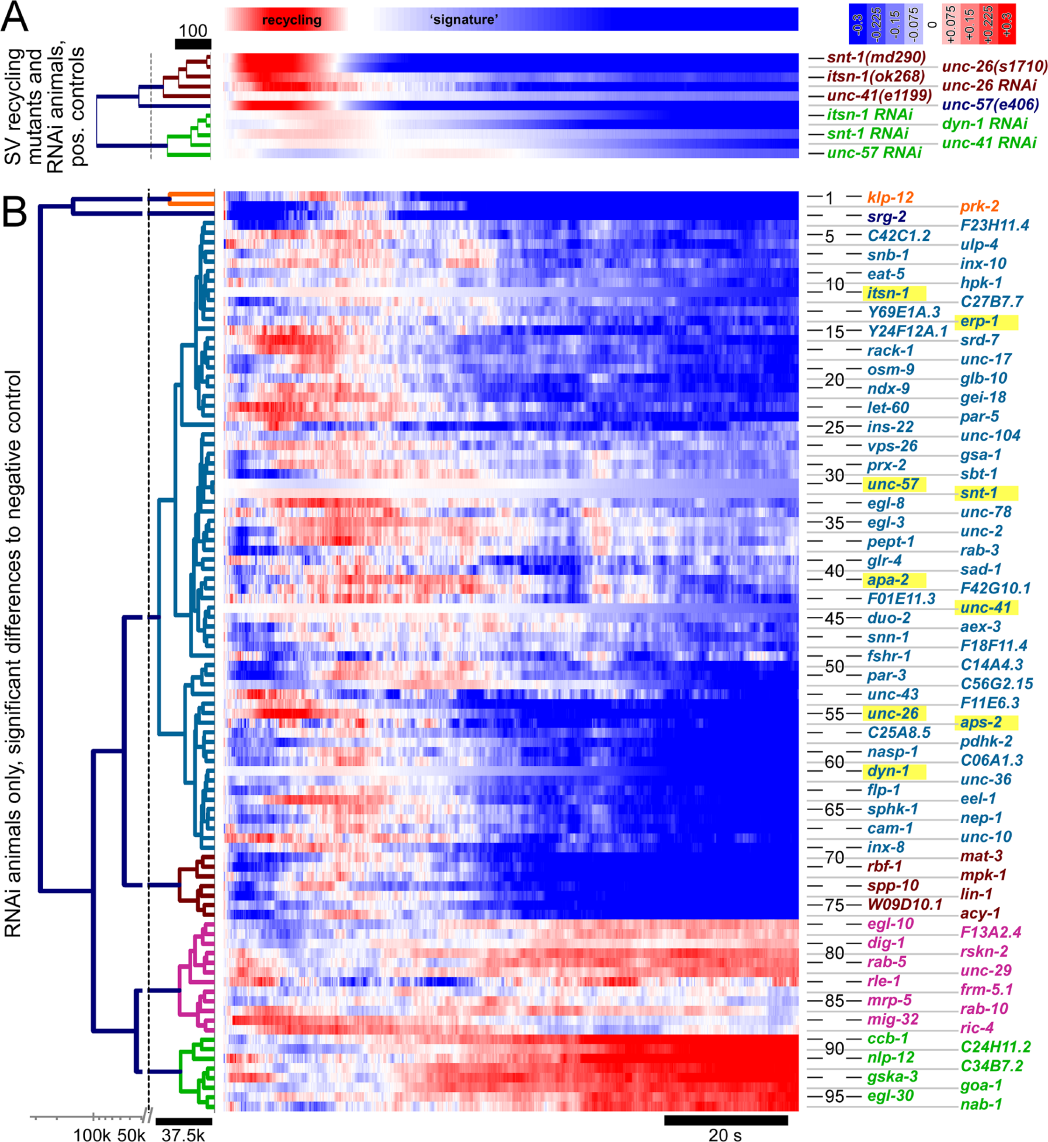
Cluster analysis of 96 genes that showed significantly altered Ca^2+^ dynamics in the R-OptIoN assay, following RNAi, or of control genomic mutants, with particular attention to potential SV-recycling phenotypes. **A)** Positive controls, i.e. mutants of known SV recycling genes, and respective RNAi animals, were sorted by agglomerative hierarchical clustering (left, distance scale is indicated); genes/RNAis are identified on the right. **B)** Of the 96 genes with significant differences to controls (2-way ANOVA with Bonferroni correction, p<0.05), 85 were previously shown to induce aldicarb resistance following systemic RNAi, and/or were included as positive controls, i.e. genes known or expected to affect SV-recycling or endocytosis (yellow shade, genes are identified on the right; see also **S1 Table**). 11 random controls were also included. Data for all 96 genes are shown as color coded table (center; color scale indicated in A, as well as the ‘SV recycling signature’), ordered by hierarchical clustering into six classes (orange, dark blue, pale blue, brown, pink and green), as indicated on the left; distance scale of the cluster analysis is shown on the lower left, note different scales, i.e. linear and logarithmic, demarcated by a broken axis. Each line represents the difference trace, obtained from peak-normalized Ca^2+^ signals, averaged from n = 3–5 biological replicates of RNAi of the respective gene (with ~1000 animals each) and the same number of biological replicates for the mock RNAi controls. Genes showing a ‘SV recycling signature’ cluster with *unc-26* and other SV recycling genes in class 1 (pale blue typing).

The data obtained for all genes was first analyzed for significant changes compared to the respective ‘empty’ RNAi feeding vector control. Of the 106 genes, 10 did not meet significance (2-way ANOVA with Bonferroni correction), as noted in **S1 Table**. The data for the 96 genes that showed significantly altered Ca^2+^ dynamics upon RNAi knockdown, are summarized in **Fig 6B** (middle panels, color coded representation; gene identity shown on the right), and the data for the positive control SV-recycling mutants we included is shown in **Fig 6A**, compared to the respective RNAi animals. We used hierarchical clustering to sort the genes according to observed phenotypes (**Fig 6**, left). The mutants and respective RNAi strains of the known SV recycling genes (**Fig 6A**) essentially clustered in two classes, where genomic mutants and RNAi animals were separated, apart from *unc-26* (synaptojanin) RNAi, which was very similar to the *unc-26(s1710)* mutant. *unc-57(e406)* endophilin was sorted between the two clusters. The RNAi knockdowns (**Fig 6B**) were clustered into 6 groups. The largest group (66 genes), class #1 (**S1 Table**), contained all the known SV recycling genes that we included (*itsn-1*, *snt-1*, *unc-26*, *unc-41*, and *unc-57*), as well as other endocytosis genes like adapter protein complex 2 components *apa-2* [67] and *aps-2* (σ2 subunit) [68], endophilin-related protein (*erp-1*) and dynamin *dyn-1*. Based on the clustering algorithm used, 57 additional genes in class #1 exhibited a Ca^2+^ dynamics signature that resembled the ‘SV recycling signature’, i.e. earlier peak Ca^2+^ signals and earlier onset of fatigue.

This included genes whose known or inferred function conceivably may contribute in the context of SV recycling, like *vps-26* (a vacuolar sorting protein homolog, part of the retromer complex [69]), *rack-1* (receptor of activated C Kinase, involved in actin cytoskeleton remodeling [70]), *snb-1* (synaptobrevin) SNARE, *snn-1* (synapsin, binding SVs to the actin cytoskeleton and to each other), *unc-10* (RIM), *egl-8* (phospholipase C β, known to promote SV priming [71, 72]), *unc-43* (CaM kinase II), *sad-1* (Ser-Thr kinase, involved in presynaptic development and SV clustering [73]), *unc-2* and *unc-36* (voltage gated Ca^2+^ channel α1 and α2/δ subunits, respectively). In addition, numerous kinases and phosphatatses, and proteins involved in ubiquitination were found in this class, whose possible role in SV recycling (in addition to the inferred role in synaptic transmission, based on aldicarb resistance) is not presently clear. Another group of proteins whose knockdown caused *unc-26*-like Ca^2+^ traces were three innexin gap junction subunits [74], *inx-8*, *inx-10* [75] and *eat-5*; the possible role of innexins in the SV cycle is not clear at present.

### R-OptIoN analysis of select genes from the SV recycling cluster in genomic mutants and by electrophysiology

**Fig 7:**
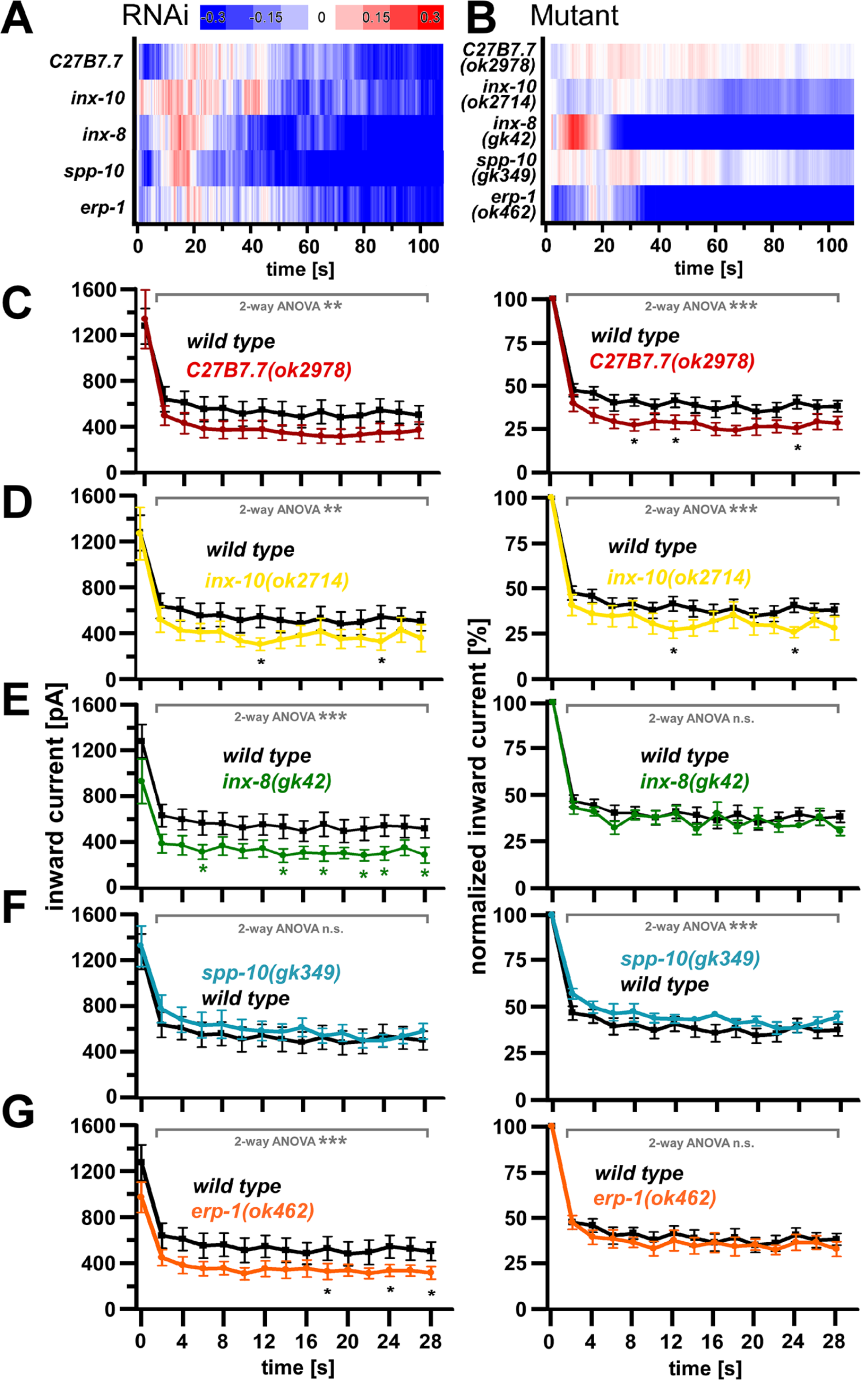
Further analysis of selected genes from the RNAi screen cluster #1, by Ca^2+^ imaging (A, B) and by electrophysiology (C-G). The genomic mutants *C27B7*.*7(ok2978)*, *inx-10(ok2714)*, *inx-8(gk42)*, *spp-10(gk349)* and *erp-1(ok462)* were crossed either to the ChR2(C128S); RCaMP strain for Ca^2+^ imaging, or to the *zxIs6* ChR2(H134R) strain, for measuring photo-ePSCs. In A and B, normalized Ca^2+^ signal difference traces are shown as color coded, maximum normalized data, based on the mean ΔF/F_0_ Ca^2+^ response (± SEM) from bulk measurements of RNAi or mutant and control (n = 3–5 experiments, ~1000 animals each). In E—G, the averaged photo-ePSC measurements of the respective wild type animals (black), *C27B7*.*7(ok2978)* (red), *inx-10(ok2714)* (yellow), *inx-8(gk42)* (green), *spp-10(gk349)* (blue), or *erp-1(ok462)* mutants (orange) are shown as inward currents in pA ± SEM (left panels). Statistically significant differences were calculated by one-way ANOVA for individual time points, always compared to wild type (* P < 0.05), or for the whole data set as two-way ANOVA (** P < 0.01, **P<0.001). Animals were stimulated for 10 ms with blue light at a frequency of 0.5 Hz (n = 7–12). On the right, the same data are represented, but normalized to the mean peak currents of the first stimulus. For representative original current records, as well as for mini ePSC current and frequency analysis, see **S5 Fig.**

Based on our RNAi screen, we found several previously unstudied genes in the *unc-26* cluster (#1), which may be involved in SV recycling. Thus, we wanted to analyze (a small number of) these genes also in genomic mutants. This should provide an indication if following up on the remaining genes from class #1 will be worthwhile in a future endeavor. Interestingly, three innexin (invertebrate gap junction) subunits were among this group, indicating that this class of proteins may be involved in synaptic recycling in a more general manner. We thus further analyzed this gene class in genomic mutants. We chose *inx-8(gk42)* and *inx-10(ok2714)*, as these genes are expressed in neurons [74]. Furthermore, we chose to study *erp-1(ok462)*, which encodes endophilin B, but for which no clear involvement in *C*. *elegans* SV recycling has been demonstrated previously. Last, we also included two additional genes, i.e. the prosaposin homologue *spp-10(gk349)* (required for normal turnover of glyco-(sphingo)-lipids), as well as *C27B7*.*7(ok2978)*, encoding a titin-like protein, as their Ca^2+^ signatures resembled that of *unc-26* synaptojanin. R-OptIoN data for the RNAi experiments is shown again in **Fig 7A**, emphasizing that for all genes, a (mild) ‘SV recycling signature’ could be detected. The mutant genes were crossed into our Ca^2+^ imaging reporter strain and analyzed for their Ca^2+^ dynamics upon photostimulation (**Fig 7B**). Furthermore, we performed electrophysiological analyses following repeated photostimulation, using the *zxIs6* transgene (punc-17::ChR2, expressed in cholinergic neurons; **Fig 7C–7G**). Based on Ca^2+^ imaging, only the *inx-8(gk42)* mutant had a strong SV-recycling signature, while the other mutants showed, if at all, only weak hints of an involvement in SV recycling (**Fig 7B**). In electrophysiological experiments, a mixed outcome was observed. Based on the absolute photo-evoked postsynaptic currents, following repeated stimulation at 0.5 Hz, all mutants apart from *spp-10* had significantly lower currents, based on 2-way ANOVA, and *inx-10*, *inx-8* as well as *erp-1* mutants also showed significantly lower currents for single time points. When looking at the currents after normalization to the first evoked peak current, to better follow progressive reduction of the evoked currents, *C27B7*.*7*, *inx-10* and *spp-10* mutants showed significantly reduced currents. However none of the mutants showed a progressive exacerbation, as was observed for *unc-26(s1710)* mutants (**Fig 3**). Thus, none of the five genes we analyzed in more detail exhibited an obvious SV recycling phenotype based on the electrophysiological assays, despite a clear SV recycling signature in Ca^2+^ imaging observed for *inx-8(gk42)* mutants.

## Discussion

Here we explored a new strategy to investigate synaptic transmission in *C*. *elegans*. We aimed to quickly detect genes required for SV recycling among factors that were previously shown to affect aldicarb sensitivity in cholinergic neurons [25]. Ca^2+^ imaging based on RCaMP in muscle cells, combined with photostimulation of cholinergic neurons using ChR2, allows analyzing how synaptic ACh release and the respective muscle response develop in a time-dependent manner. This method is equivalent to our previously used behavioral readout, i.e. following muscle contraction by video microscopy [14, 28]. Yet, it is better suited for large-scale screening purposes based on RNAi, due to the simultaneous analysis of ~1000 animals, when compared to contraction assays, where single animals need to be analyzed, largely increasing the workload. A previous attempt to achieve parallelization in microfluidic channels proved to be too unreliable [29].

Analyses of characterized genes affecting synaptic transmission allowed defining a ‘signature’ of the developing Ca^2+^ transients for mutants affecting SV biogenesis by endocytic recycling (endophilin A *unc-57*, synaptojanin *unc-26*, intersectin *itsn-1*, stonin *unc-41*, synaptotagmin *snt-1*, Eps15 *ehs-1* and dynamin *dyn-1*), or for generation of functional SVs, e.g. filling of SVs with ACh (vAChT *unc-17*). This signature allowed distinguishing genes among the 96 genes involved in synaptic transmission that we tested, as they showed a similar signature, based on hierarchical clustering of the data. All of the known SV recycling or endocytosis genes were among these genes in cluster #1. This could provide a first indication that the respective proteins may affect SV recycling or another rate-limiting step in their biogenesis, or could be involved in exocytosis-endocytosis coupling. However, when comparing mutant phenotypes to RNAi induced phenotypes, only *unc-26* synaptojanin RNAi was able to induce a similarly strong recycling signature as the genomic mutant. Additionally, among the genes from cluster #1 which we then further analyzed in genomic mutants, only the innexin *inx-8* had a strong recycling signature. Unfortunately, further analysis by electrophysiology could not confirm a SV recycling phenotype for this gene.

In sum, the results from RNAi-induced depletion of the respective mRNA in cholinergic neurons, and the phenotypes observed in genomic mutants, did not always match, and RNAi phenotypes were in most cases weaker than the mutant phenotypes. Thus, how robust is the Ca^2+^ imaging ‘SV recycling signature’? Based on analyses of the post-synaptic Ca^2+^ dynamics for established, ‘strong’ SV recycling mutants, like *unc-26*, our approach is robust. Thus, it could, in principle, allow identifying genes with strong recycling defects even in a genome-wide RNAi screen, while it will likely not be able to detect genes causing subtle SV recycling defects. Therefore, stringent criteria for inclusion in secondary screening have to be used. Here we tested the approach on genes that were previously shown to have aldicarb sensitivity defects. The genes as identified by Sieburth et al. [25] were isolated from a rather limited number of ca. 2000 pre-selected genes. Thus, if there are strong SV recycling factors in the remainder of the genome, our approach may allow identifying them. In particular, it would enable to search among factors whose loss in tissues other than cholinergic neurons, or during development, could induce lethality or growth arrest, which would not be identified in forward genetic or in systemic RNAi screens. For a genome-wide screen, however, further automatization would be required, for example using a modified plate reader [76] and 96-well format for imaging of the animals, and cultivation in this format in liquid culture. The low efficiency of RNAi in inducing SV recycling signatures for genes showing strong signatures as mutants (apart from *unc-26*), however, makes it questionable if a genome-wide screen is really feasible.

Comparing cholinergic neuron specific RNAi experiments and genomic mutants might have resulted in different phenotypes because a mutant will affect the gene in other tissues as well, such that masking phenotypes may add to the cholinergic neuron specific phenotype. A recent approach to use recombinase-based transgene rescue in specific tissues, in the mutant background, may allow to look at phenotypes of a genomic mutant in cholinergic neurons [77]. Alternatively, RNAi depletion may have more severe phenotypes than the genomic mutants of the respective genes that were available, as these alleles may have subtle reduction of function phenotypes or could be hypomorphs rather than loss-of-function alleles. Interpretation of phenotypes is further complicated by the sometimes overlapping function of genes in the many steps of the SV cycle. However, our approach allowed distinguishing characteristics of mutants described as defective in pre- and post-synaptic transmission and the different steps in the SV cycle. Defective pre-synaptic Ca^2+^ signaling in *unc-2(ra612)* mutants caused an overall increased Ca^2+^ response in muscle, with otherwise normal Ca^2+^ dynamics. Defective transmitter release (*snt-1(md290)* synaptotagmin), or post-synaptic transmitter sensing (*unc-38(x20)* nAChR subunit) led to an overall weaker Ca^2+^ response. Mutants defective for synaptic vesicle recycling (*unc-57* endophilin, *unc-26* synaptojanin, *itsn-1* intersectin, and to a lesser extent, *unc-41* stonin, as well as *ehs-1* Eps15) had the unique characteristic of beginning with a stronger activation, followed by a characteristic appearance of fatigue. This fatigue was also seen in mutants impairing vesicle loading (*unc-17(e113)* vAChT), or synaptotagmin, which acts as a clathrin adaptor in SV recycling, in addition to its role in release [78]. Thus, through R-OptIoN it is possible to get a first hint for a particular protein’s involvement in different steps of the SV cycle.

## Supporting Information

S1 Fig Preparation of micro-wells for bulk Ca^2+^ fluorescence measurements. Agar pads of the thickness of one microscope slide were poured. A pre-heated LED cooling element was used to stamp wells into the agar pad.
(TIF)

S2 Fig Workflow of Ca^2+^ imaging data analysis. (PDF)

S3 Fig Top row: Fluorescence images from single frames of a time-lapse RCaMP Ca^2+^ measurement in body wall muscles of ~1000 animals, inside a measurement well, during photostimulation of cholinergic motoneurons (expressing ChR2). Single representative images from the indicated time points from the video stream are shown in a color look-up table. Bottom: Each bar represents the mean intensity of the representative images (whole field of view), beginning at the first frame during stimulation, until 33s during stimulation.
(TIF)

S4 Fig Contraction profiles of animals expressing ChR2(C128S)::YFP in cholinergic motoneurons during ChR2 activation (blue bar marks period of blue light stimulation). To mimic experimental conditions during RCaMP imaging, this experiment was performed under additional, constant 590 nm illumination (orange bar). Shown are mean normalized body length (± SEM) for wild type (black trace), no-ATR control (grey trace), and *unc-26(s1710)* (synaptojanin, red trace) (n = 7–36).
(TIF)

S5 Fig Mutants analyzed in this study (controls, as well as novel putative SV recycling genes) were assessed by patch clamp recordings from body wall muscles, downstream of cholinergic motoneurons that were photostimulated using ChR2. **A)** Original records of inward currents following repeated photostimulation (0.5 Hz), mutant strains as indicated. **B)** Baseline, mean (±SEM) miniature post synaptic current events (mPSCs per second) from the indicated number of animals (genetic background as labeled), during a period before photostimulation. **C)** Mean mPSC amplitudes of the same strains as in B. Statistically significant differences to wild type (one-way ANOVA) are indicated (* P< 0.05; ** P < 0.01).
(TIF)

S6 Fig Mutants in *ehs-1(ok146)* and *dyn-1* RNAi animals were compared to *unc-26(s1710)* and *unc-26* RNAi animals for their Ca^2+^ traces (color coded representation after maximum normalization of the mean data). (TIF)

S1 Table Genes assessed in the RNAi screen, listed according to the sorting into six clusters by hierarchical clustering, as shown in Fig 6, ordered using the RNAi/Ca^2+^ imaging results. Class 1 contains genes that cluster with *unc-26* synaptojanin and other SV recycling positive controls (yellow shading). Color shading is explained in the legend. Genes from the Sieburth et al. (2005) RNAi screen are shown in white, if they were only shown to induce aldicarb resistance following systemic RNAi. Light grey indicates genes that were also verified in genomic mutants by aldicarb assays. Orange shading represents random picked ‘negative’ controls. Blue, green and pink shading includes additional genes inducing aldicarb phenotypes, or genes that were lethal upon systemic RNAi. Genes that were analyzed by RNAi, but did not show significantly different Ca^2+^ traces compared to controls (based on 2-way ANOVA with Bonferroni correction), are indicated on the lower right.
(PDF)

S1 Video A video obtained from a micro-well, filled with animals expressing RCaMP in body wall muscle, before and during the blue-light stimulus phase. First, a jump in RCaMP fluorescence is seen when the ChR2 stimulation light is turned on, followed by a further, slower increase in fluorescence while muscles are stimulated by ongoing cholinergic neuron activity.
(AVI)

S1 Data This .rar archive contains files “Anova 1”, “Anova 2” and “Positive controls”, including Ca^2+^ imaging data (mean, base-line corrected fluorescence of the ROI depicting the micro-well), shown for each RNAi strain in the genetic screen, for each biological replicate, as well as the respective control imaging data traces (animals treated with empty RNAi feeding vector) for the respective RNAi experiment. Videos were obtained at 20 fps, reflected by the ~2400 data points obtained for each trace during each ~130 s experiment. Furthermore, the peak normalizations are calculated in the data tables, as well as the 2-way ANOVA comparisons.
(RAR)

S2 Data This .rar archive contains files “RNAi 1–30” and “RNAi 31–60”, including Ca^2+^ imaging data (mean, base-line corrected fluorescence of the ROI depicting the micro-well), shown for each RNAi strain in the genetic screen, for each biological replicate, as well as the respective control imaging data traces (animals treated with empty RNAi feeding vector) for the respective RNAi experiment. Videos were obtained at 20 fps, reflected by the ~2400 data points obtained for each trace during each ~130 s experiment. Furthermore, the peak normalizations are calculated in the data tables.
(RAR)

S3 Data This .rar archive contains files “RNAi 61–90” and “RNAi 91–105”, including Ca^2+^ imaging data (mean, base-line corrected fluorescence of the ROI depicting the micro-well), shown for each RNAi strain in the genetic screen, for each biological replicate, as well as the respective control imaging data traces (animals treated with empty RNAi feeding vector) for the respective RNAi experiment. Videos were obtained at 20 fps, reflected by the ~2400 data points obtained for each trace during each ~130 s experiment. Furthermore, the peak normalizations are calculated in the data tables.
(RAR)
