# Supplementary figures and images for: High-Throughput All-Optical Analysis of Synaptic Transmission and Synaptic Vesicle Recycling in Caenorhabditis elegans
Source: PLoS One. 2015 Aug 27;10(8):e0135584. doi: 10.1371/journal.pone.0135584 (PMC4552474; doi:10.1371/journal.pone.0135584)

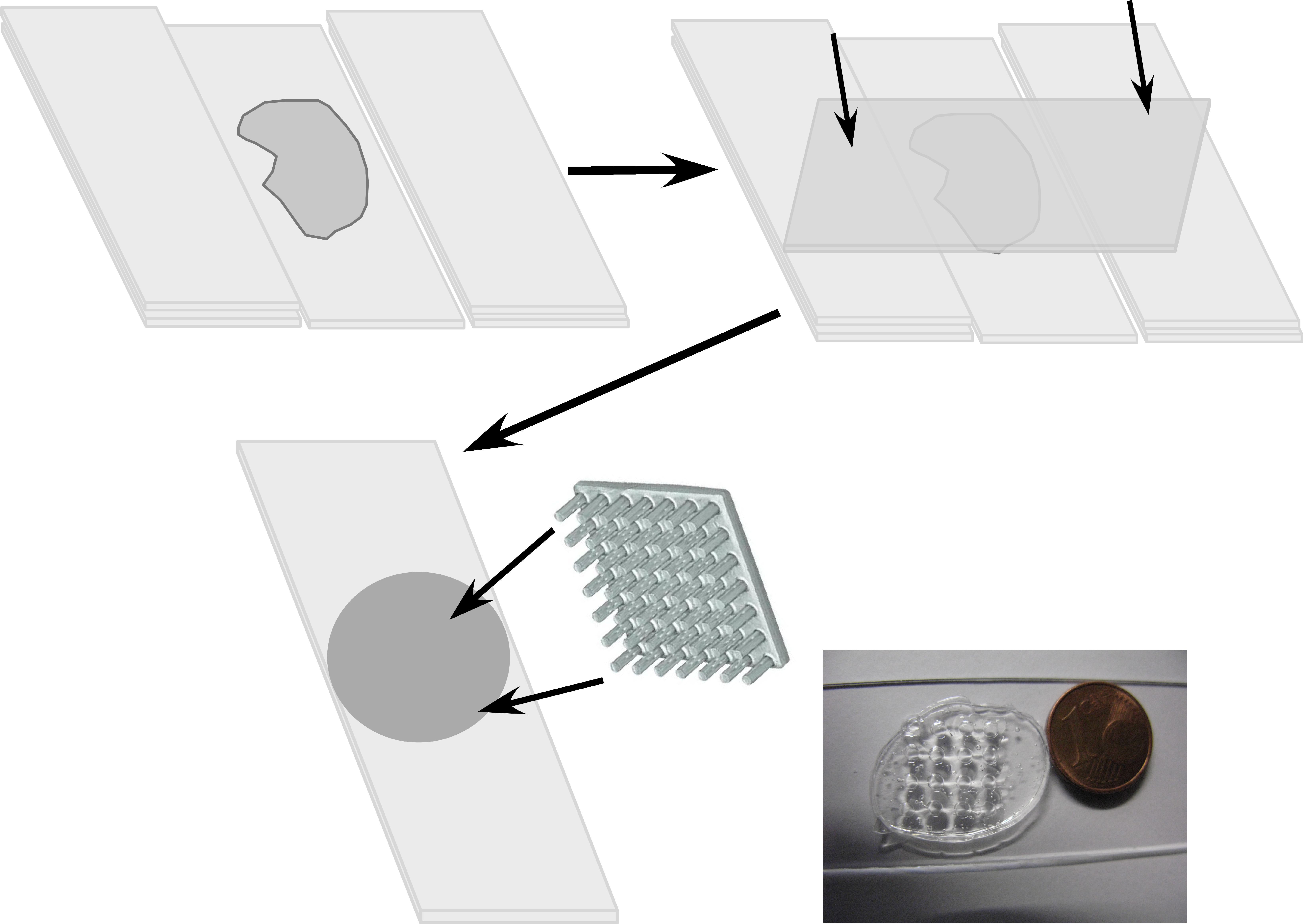

Supplement: S1 Fig — Agar pads of the thickness of one microscope slide were poured. A pre-heated LED cooling element was used to stamp wells into the agar pad. (TIF) [file pone.0135584.s001.tif]

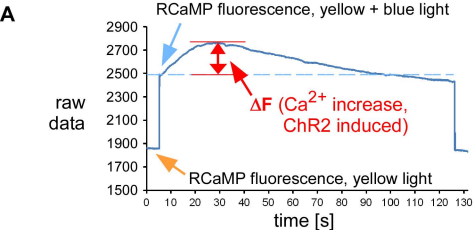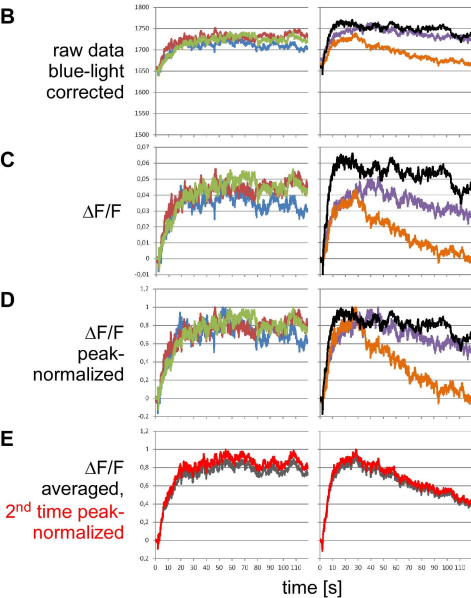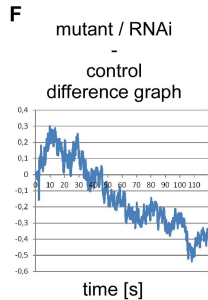

Supplement: S2 Fig — (PDF) [file pone.0135584.s002.pdf]

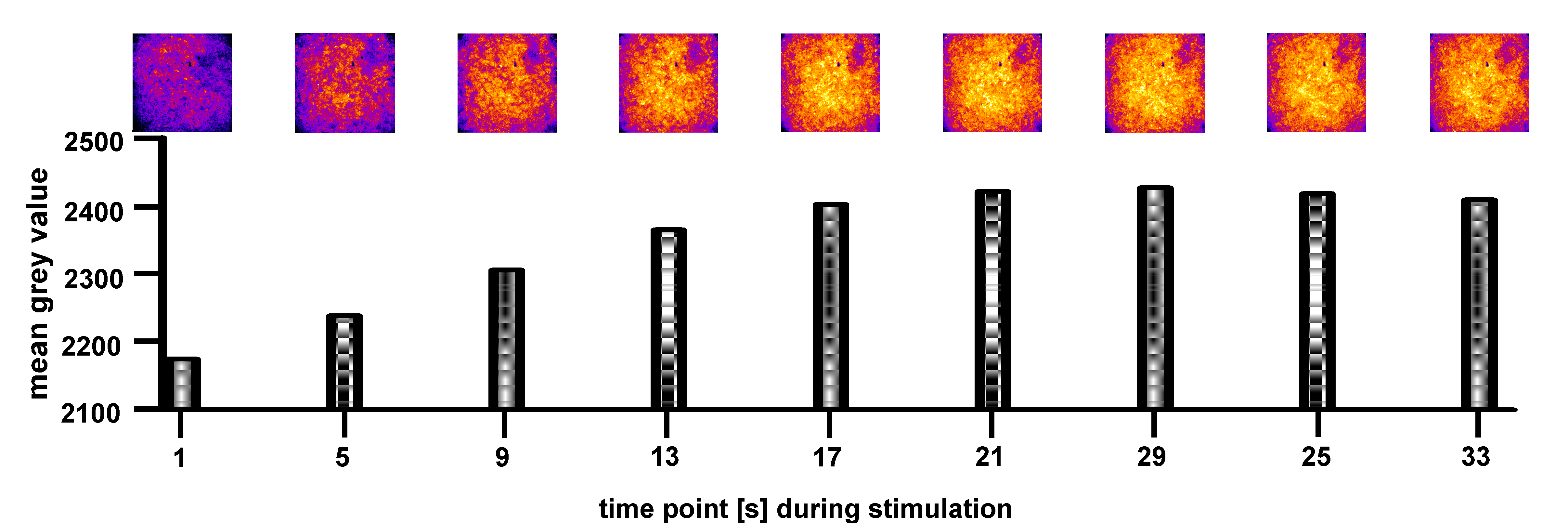

Supplement: S3 Fig — Single representative images from the indicated time points from the video stream are shown in a color look-up table. Bottom: Each bar represents the mean intensity of the representative images (whole field of view), beginning at the first frame during stimulation, until 33s during stimulation. (TIF) [file pone.0135584.s003.tif]

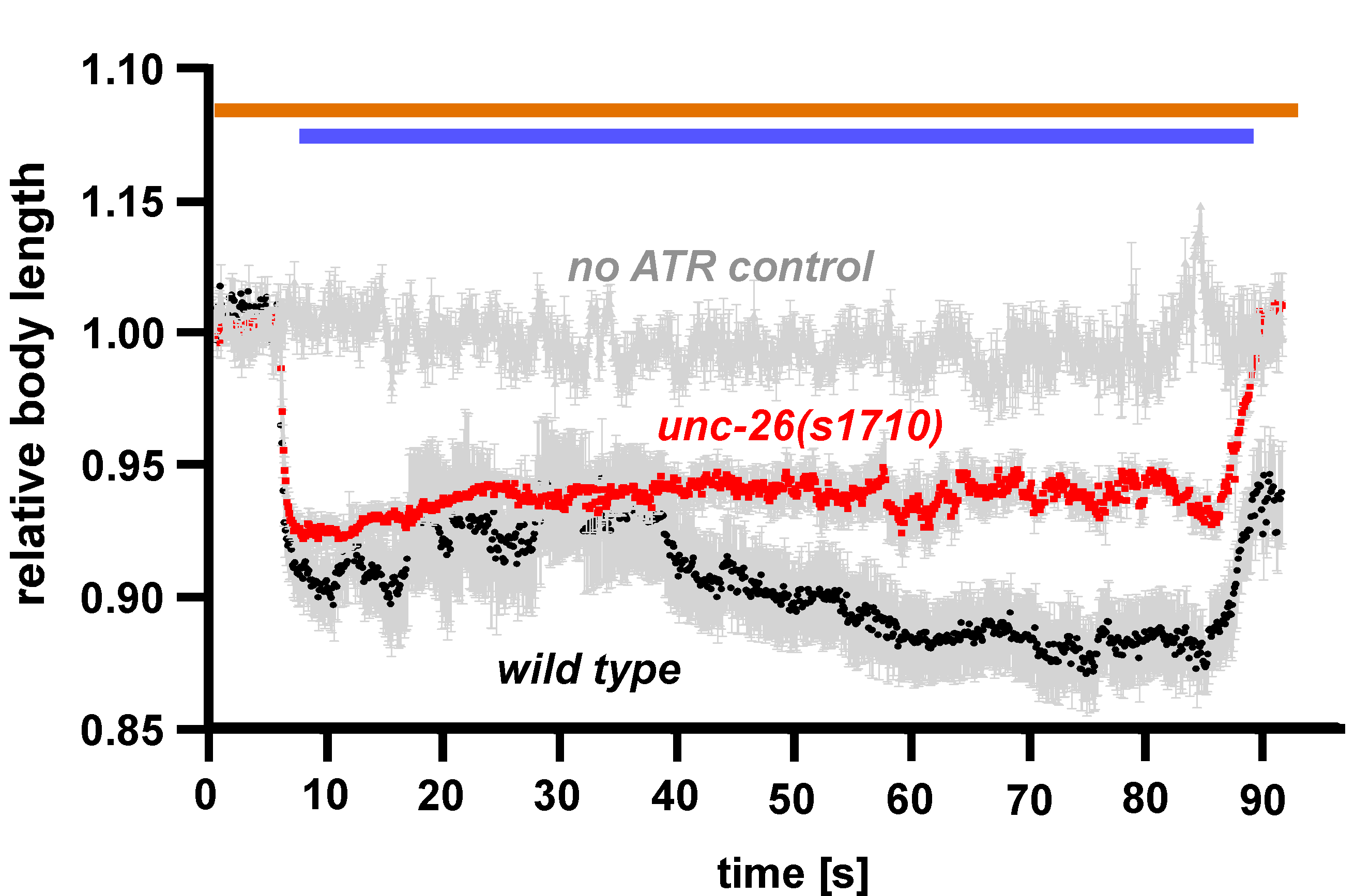

Supplement: S4 Fig — To mimic experimental conditions during RCaMP imaging, this experiment was performed under additional, constant 590 nm illumination (orange bar). Shown are mean normalized body length (± SEM) for wild type (black trace), no-ATR control (grey trace), and unc-26(s1710) (synaptojanin, red trace) (n = 7–36). (TIF) [file pone.0135584.s004.tif]

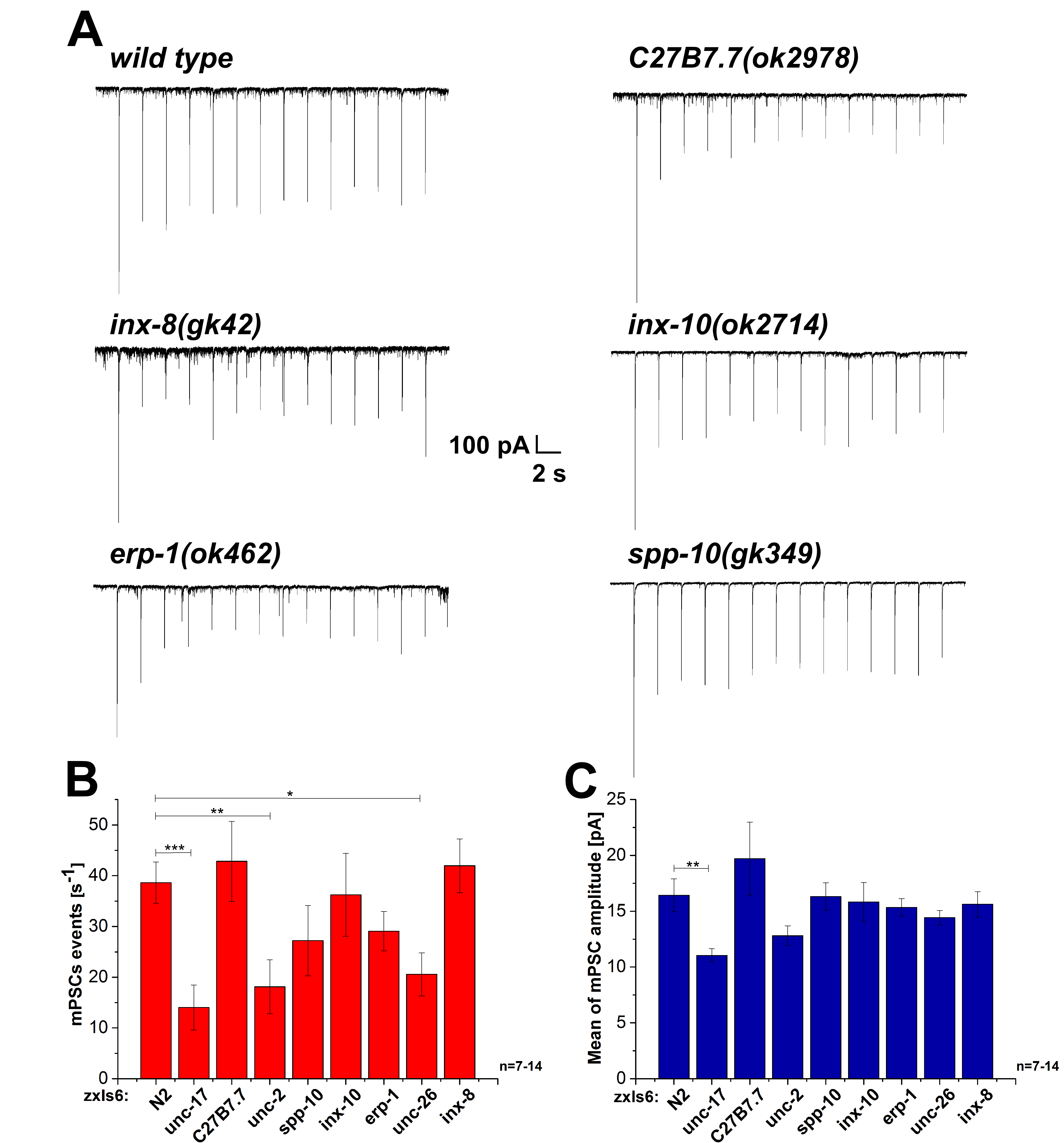

Supplement: S5 Fig — A) Original records of inward currents following repeated photostimulation (0.5 Hz), mutant strains as indicated. B) Baseline, mean (±SEM) miniature post synaptic current events (mPSCs per second) from the indicated number of animals (genetic background as labeled), during a period before photostimulation. C) Mean mPSC amplitudes of the same strains as in B. Statistically significant differences to wild type (one-way ANOVA) are indicated (* P< 0.05; ** P < 0.01). (TIF) [file pone.0135584.s005.tif]

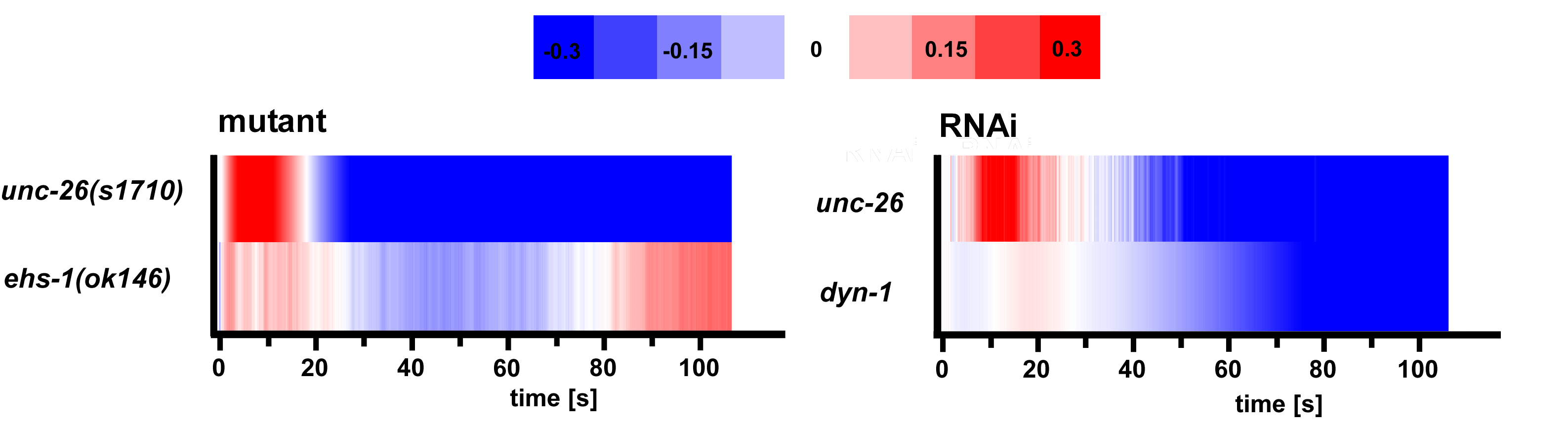

Supplement: S6 Fig — (TIF) [file pone.0135584.s006.tif]
