# Supplementary material for: High-Throughput All-Optical Analysis of Synaptic Transmission and Synaptic Vesicle Recycling in Caenorhabditis elegans
Source: PLoS One. 2015 Aug 27;10(8):e0135584. doi: 10.1371/journal.pone.0135584 (PMC4552474; doi:10.1371/journal.pone.0135584)
Supplement: S1 Table — Class 1 contains genes that cluster with unc-26 synaptojanin and other SV recycling positive controls (yellow shading). Color shading is explained in the legend. Genes from the Sieburth et al. (2005) RNAi screen are shown in white, if they were only shown to induce aldicarb resistance following systemic RNAi. Light grey indicates genes that were also verified in genomic mutants by aldicarb assays. Orange shading represents random picked ‘negative’ controls. Blue, green and pink shading includes additional genes inducing aldicarb phenotypes, or genes that were lethal upon systemic RNAi. Genes that were analyzed by RNAi, but did not show significantly different Ca2+ traces compared to controls (based on 2-way ANOVA with Bonferroni correction), are indicated on the lower right. (PDF) [file pone.0135584.s007.pdf]

Genes chosen for RCaMP/RNAi screen, based on Sieburth et al., 2005, and controls

sorted by agglomerative hierachical clustering into 6 classes (Manhattan distance, complete linkage)

#	class 1	
1	*ins-22*	insulin like neuropeptide
2	*sbt-1*	prohormone convertase
3	*rab-3*	rab3, SV priming
4	*fshr-1*	neuropeptide receptor
5	*sad-1*	Ser/Thr protein kinase
6	*glr-4*	ionotropic glutamater receptor
7	*unc-43*	CaMKII
8	*Y24F12A.1*	putative deoxyribonuclease
9	*inx-10*	Innexin, gap junction
10	*vps-26*	retromer complex
11	*rack-1*	receptor of activated C kinase
12	*let-60*	RAS
13	*unc-26*	synaptojanin
14	*par-5*	14-3-3 protein
15	*F11E6.3*	brain acid insoluble protein 1
16	*srd-7*	GPCR
17	*aps-2*	adaptin, sigma2 subunit of AP2
18	*inx-8*	Innexin, gap junction
19	*ndx-9*	NUDIX hydrolase
20	*gei-18*	GEX interacting protein
21	*C42C1.2*	protein phosphatase
22	*hpk-1*	Protein kinase
23	*pdhk-2*	pyruvate dehydrogenase kinase
24	*unc-36*	VGCC alpha2/delta subunit
25	*eat-5*	Innexin, gap junction
26	*nep-1*	Neprilysin metallopeptidase
27	*C06A1.3*	Ser/Thr protein phosphatase
28	*sphk-1*	Sphingosine kinase
29	*cam-1*	receptor tyrosine kinase
30	*nasp-1*	Nuclear autoantigenic sperm prot.
31	*ulp-4*	Ubiquitin-like protease
32	*erp-1*	endophilin B
33	*eel-1*	Hect E3 ubiquitin ligase

#	class 1	
34	*unc-10*	RIM (rab-3 interacting molecule)
35	*glb-10*	globin
36	*F18F11.4*	unknown
37	*aex-3*	rab-3 GEF
38	*Y69E1A.3*	Tyrosine-protein kinase
39	*C56G2.15*	putative N-acetyltransferase
40	*duo-2*	deubiquinating protein
41	*pept-1*	oligopeptide transporter
42	*unc-2*	vGCC alpha subunit
43	*prx-2*	peroxisomal membrane protein
44	*egl-3*	proprotein convertase
45	*F42G10.1*	neprilysin metallopeptidase
46	*apa-2*	adaptin complex alpha chain
47	*F23H11.4*	Arf-GAP with Rho-GAP domain
48	*C27B7.7*	Titin-like protein
49	*par-3*	PDZ domain, polarization
50	*C14A4.3*	glycosyltransferase
51	*snb-1*	synaptobrevin
52	*egl-8*	phospholipase C beta
53	*flp-1*	FRMF neuropeptide precursor
54	*C25A8.5*	Tyrosine-protein kinase Fer
55	*gsa-1*	G alpha S
56	*osm-9*	TRPV channel
57	*snn-1*	synapsin
58	*unc-17*	vAChT
59	*unc-104*	kinesin
60	*F01E11.3*	unknown
61	*dyn-1*	dynamin
62	*itsn-1*	intersectin
63	*snt-1*	synaptotagmin
64	*unc-41*	stonin
65	*unc-57*	endophilin A
66	*unc-78*	actin-interacting protein

#	class 2	
67	*rle-1*	RING finger E3 ubiquitin ligase
68	*unc-29*	nAChR subunit
69	*rab-5*	RAB GTPase, endosome
70	*F13A2.4*	unknown
71	*rskn-2*	ribosomal protein S6 kinase a-2
72	*dig-1*	immunoglobulin superfamily
73	*egl-10*	RGS for GOA-1
74	*mrp-5*	ABC transporter, CFTR
75	*rab-10*	RAB GTPase, endocytosis
76	*frm-5.1*	FERM domain, scaffold protein
77	*ric-4*	SNAP-25
78	*mig-32*	Polycomb repressive complex 1

#	class 3	
79	*C24H11.2*	Ser/Thr protein phosphatase
80	*nlp-12*	neuropeptide
81	*gska-3*	Glycogen Synthase Kinase alpha
82	*C34B7.2*	Phosphoinositide phosphatase
83	*goa-1*	G alpha O
84	*nab-1*	Neurabin/Spinophilin protein
85	*ccb-1*	VGCC beta subunit
86	*egl-30*	G alpha Q

#	class 4	
87	*rbf-1*	Rabphilin
88	*mat-3*	CDC23/APC8, APC/C regulator
89	*lin-1*	transcription factor
90	*acy-1*	adenylyl cyclase
91	*spp-10*	prosaposin
92	*W09D10.1*	membrane, ARF GAP
93	*mpk-1*	MAP kinase

#	class 5	
94	*prk-2*	kinase
95	*klp-12*	kinesin like protein

#	class 6	
96	*srg-2*	GPCR

did not meet significance (2-way ANOVA vs. neg. control)	
inx-15	Innexin, gap junction
unc-18	SV priming factor
usp-46	Ubiquitin specific protease
let-70	E2 ubiquitin conjugating enzyme
arx-3	Arp2/3 complex component
wdr-23	ubiquitin ligase complex
pkc-1	Protein kinase C
fer-1	Ferlin, membrane fusion
tbc-7	Tre-2/Bub2/Cdc16 domain family
tag-232	PACS1 sorting protein

unc-26 (reference SV recycling gene)
aldicarb resistant, systemic RNAi, Sieburth et al., 2005
aldicarb resistant, also in mutants; Sieburth et al., 2005
lethal in systemic RNAi, Sieburth et al., 2005

other aldicarb resistant genes (www.wormbase.org)
GABA aldicarb RNAi phenotypes; Vashlishan et al., 2008
random controls
RNAi positive controls of other known SV recycling genes
